# Vitamin A-containing dietary supplements from German and US online pharmacies: market and risk assessment

**DOI:** 10.1007/s00210-024-03050-6

**Published:** 2024-03-28

**Authors:** Anna-Miriam Rathmann, Roland Seifert

**Affiliations:** Institute of Pharmacology, Hannover Medical School, D-30625 Hannover, Germany

**Keywords:** Vitamin A, Vitamin A intake, Vitamin A supplements, Dietary supplements Germany, Dietary supplements USA, Pubmed.gov

## Abstract

Vitamin A supplements are used by many people, and the number of newly registered dietary supplements is continuously increasing. The preparations fall under food law and are not subject to the strict controls of pharmaceuticals. Risk indications and maximum quantity recommendations, e.g., from the Bundesinstitut für Risikobewertung (BfR) and the U.S. Food and Drug Administration (FDA) are not binding, which means that overdoses and potentially serious health problems can easily occur. The hepatotoxicity and teratogenicity of vitamin A are well documented, and other negative effects of high doses of vitamin A are also being discussed. Nevertheless, preparations with exorbitantly high doses are freely available for sale and unrestricted. In this study, 75 supplements containing vitamin A available in Germany and 26 available in the USA were critically examined on the basis of various parameters such as the recommended daily dose according to the manufacturer, daily therapy costs (DTC), the presence of warnings about overdose, use during pregnancy and breastfeeding, and information on adverse effects/interactions. The aim was to gain insights into their risk potential and to examine the need for closer monitoring and stricter guidelines for these preparations. The results show some considerable country-specific differences. Overall, there are serious deficiencies in compliance with the labeling requirements for both the German and the US preparations, and the dosages are often far too high in view of the applicable expert recommendations. Overall, these deficits can pose a risk for consumers that is difficult to assess in its entirety, especially for vulnerable consumer groups. It should be noted that the US preparations perform better overall than the German preparations. This suggests better regulation of dietary supplements in the US market. Based on the available data and literature research, it is doubtful whether the intake of vitamin A-containing preparations, without a diagnosed vitamin A deficiency, has a positive health benefit. Furthermore, it should be examined whether vitamin A should continue to be offered over-the-counter as a food supplement.

**Supplementary Information:**

The online version contains supplementary material available at 10.1007/s00210-024-03050-6.

## Introduction

**Fig. 1:**
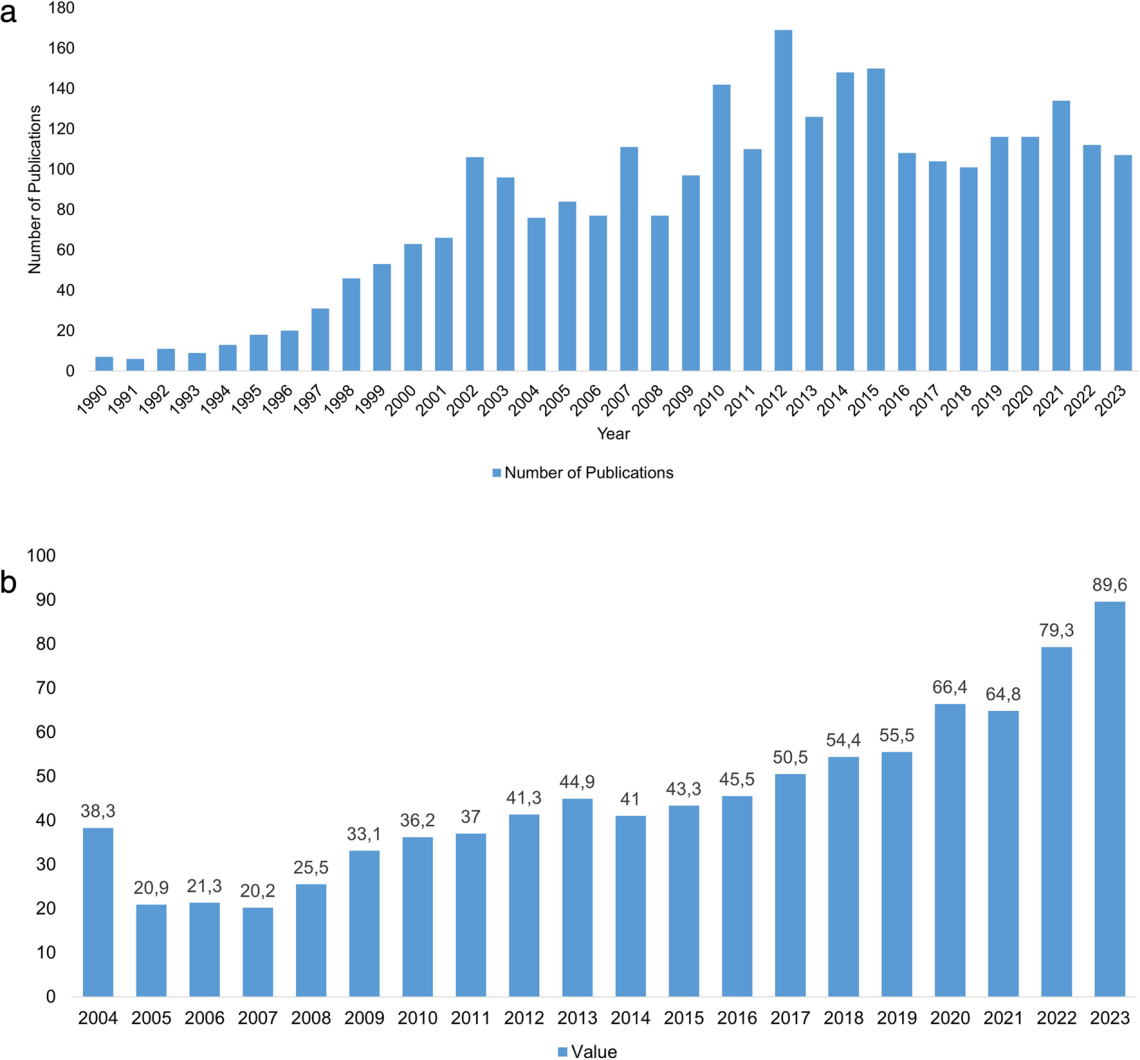
**a** Chronological development of publications for the search terms “Dietary supplements Vitamin A” on PubMed.gov (retrieved on 09.12.2023) (https://pubmed.ncbi.nlm.nih.gov/?term=dietary+supplement+vitamin+A&filter=years.1990-2023). **b** Chronological development of Google time trend for the search terms “vitamin A Supplements” on trends.google.com (retrieved on 24.02.2024). The values ​​indicate the search interest relative to the highest point in the chart for the selected region during the specified period. A value of 100 represents the highest popularity of this search term. A value of 50 means that the term is half as popular, and a value of 0 means that there was not enough data for this term. (https://trends.google.com/trends/explore?date=2004-01-01%202023-12-31&q=vitamin%20A%20supplements&hl=de)

According to a survey conducted in 2022, three out of four people in Germany take dietary supplements. Vitamins are by far in the lead here with a share of 58% (Statista Daily Data, Brandt 2022). Looking at the individual subgroups, vitamin A and vitamin D as monopreparations and combination preparations account for sales of 12.4 million packs in 2022 (Arbeitskreis Nahrungsergänzungsmittel (AK NEM) im Lebensmittelverband Deutschland e. V. 2022). The PubMed and Google Time Trend shown in Fig. 1a and b, respectively, also indicate the increasing number of publications on the topic of dietary supplements and vitamin A and the increasing number of searchings for vitamin A supplements on Google. They underline the topicality and relevance of this topic. The preparations on offer are freely available for sale and, like all dietary supplements, are subject to food law. In contrast to medicinal products, they are not subject to strict controls (NemV 2004). Responsibility for ingredients and dosage recommendations lies solely with the manufacturer. As risk indications and maximum quantity recommendations, e.g., from the Bundesinstitut für Risikobewertung (BfR), are not binding, there is a risk of overdosing, which can lead to serious health risks (Bundesinstitut für Risikobewertung (BfR) 2021a). Table 1 shows the reference values for the intake of vitamin A published by the BfR in its report “Proposed maximum levels for vitamin A in foods including dietary supplements,” the reference values for the intake of vitamin A published by the U.S. Food and Drug Administration in the Code of Federal Regulations Title 21, and the Tolerable Upper Intake Level for vitamin A established by the SCF in its Opinion on the Tolerable Upper Intake Level of Preformed Vitamin A (Retinol and Retinyl Ester) (Bfr 2021, 21 CFR § 101.^9^, SCF 2002).

**Table 1:** Recommended intakes and acceptable upper intake levels for vitamin A from the BfR report “Höchstmengenvorschläge für Vitamin A in Lebensmitteln inklusive Nahrungsergänzungsmitteln,” the Food and Drug Administration’s Code of Federal Regulations, and the Scientific Committee on Food’s “Opinion on the Tolerable Upper Intake Level of Preformed Vitamin A”

Age	Recommended daily intake (µg) of vitamin A (Bundesinstitut für Risikobewertung (BfR) 2021a)		Tolerable upper intake level (UL) vitamin A (µg) (Bundesinstitut für Risikobewertung (BfR) 2021a) and CSF 2002)	Recommended daily intake (µg) of vitamin A (U.S. Food and Drug Administration 2023b)	
	Women	Men		Women	Men
Adults 19–65 years	700 µg	850 µg	3000 µg	900 µg	900 µg
Pregnant women	800 µg			1300 µg	
Postmenopausal women	700 µg		1500 µg (Bundesinstitut für Risikobewertung (BfR) 2021a)		

The term “vitamin A” covers a family of compounds, including retinol, retinal, and retinoic acid. These substances are products of animal metabolism; while their precursor forms, the provitamins occur in plants. The different forms of vitamin A fulfill different biological functions and play a crucial role in numerous biochemical and physiological processes in the human body. For example, retinal is essential for maintaining visual function. As a precursor of the visual pigment rhodopsin in the photoreceptor cells of the retina, it is bound to rhodopsin in the form of 11-cis-retinal. When exposed to light, it is isomerized into all-trans-retinal, which leads to a conformational change in rhodopsin and triggers the onset of the visual process. Retinoic acid, in turn, is present in all tissues and plays a role in cell proliferation and differentiation. As a morphogen, it influences organogenesis during embryonic development. The gene regulatory effects of retinoic acid extend to the control of gene expression of proteins that regulate cell growth and differentiation (such as interleukins, cytokines, and oncogenes) as well as cell–cell interactions (such as fibronectin and laminin). In this way, retinoic acid exerts its effects on various tissues such as the respiratory epithelium, the intestinal mucosa, the skin, and hematopoiesis (the differentiation of myeloid cells) (Sporn et al. 1971, Dawson 2000).

Nutritional vitamin A deficiencies are found almost exclusively in countries with a low level of development (World Health Organization 2009, Zhao et al. 2022). The main symptoms include night blindness and increased sensitivity to glare, as well as keratomalacia, xerophthalmia, and atrophy of the salivary glands and the mucous membranes of the respiratory, gastrointestinal, and urogenital tracts in cases of advanced deficiency. Causes of vitamin A deficiency in people in industrialized countries include nutritional deficiencies in elderly people who are undersupplied at home, patients with chronic inflammatory bowel diseases such as Crohn’s disease and ulcerative colitis, or other digestive and absorption disorders. Liver cirrhosis, long-term alcohol, and nicotine abuse as well as short bowel syndromes, e.g., after partial small bowel resection, should also be mentioned. Genetic causes can be found in cystic fibrosis (Aktories 2013). Various studies show that the recommended daily intake of vitamin A is largely achieved in Germany and the USA (Adolf et al. 1995; Kersting et al. 2004; Zhao et al. 2022). Vitamin A intake from dietary supplements is not taken into account here. In this context, the BfR recommends avoiding the intake of additional vitamin A from dietary supplements wherever possible (Bundesinstitut für Risikobewertung (BfR) 2021a). If vitamin A-containing dietary supplements are taken, a maximum daily dose of 400 µg is recommended (Bundesinstitut für Risikobewertung (BfR) 2021a). The FDA does not make any direct recommendations on vitamin A intake via dietary supplements (U.S. Food and Drug Administration 2023b).

Among the people surveyed by the BfR, 70% stated that they felt “moderately” or “(not at all) well” informed about the health risk of vitamins as dietary supplements (Bundesinstitut für Risikobewertung (BfR) 2021b). The toxicity of vitamin A was first described in the 1940s (Herbst et al. 1944) and especially the hepatotoxicity (Biesalski 1989; Hathcock et al. 1990; Nollevaux et al. 2006) and teratogenicity (Hayes et al. 1981; Hendrickx et al. 2000; Rothman et al. 1995) are well documented. An increased risk of lung cancer with long-term use of individual β-carotene, retinol, and lutein supplements is also reported in epidemiological observational studies (Omenn et al. 1996; Satia et al. 2009), and a negative influence on bone health (Lind et al. 2017; Yorgan et al. 2016) has been discussed. The decision to take vitamin A supplements should therefore not be taken lightly.

In view of the fact that no conclusive studies are available beyond a few case reports, this study compares over-the-counter vitamin A supplements and combination preparations containing vitamin A that are available in German and US online pharmacies.

## Materials and methods

The product information is available for download on the respective website, and if available, the outer packaging of the products was used to examine the individual preparations (last retrieval date: 28.09.2023 ((DocMorris, MedPex, Shop-Apotheke; Germany); 24.10.2023 (Walgreens; USA)). The following online pharmacies were searched:

- https://www.docmorris.de/
- https://www.medpex.de/
- https://www.shop-apotheke.com/
- https://www.walgreens.com/

The retrieval data of the product information was documented in tabular form as part of the data collection. The items collected were as follows: pictures of the preparations, Pharmaceutical Central Number (PZN)/European Article Number (EAN)/Global Trade Item Number (GTIN), product name, brand, manufacturer, country of manufacture, monopreparation/combination preparation, recommended retail price (RRP) (€), and daily therapy cost (DTC) (€) (for the US online pharmacy products, the exchange rate of 02.11.2023 was used to convert from US dollars to euros, which was 1.07 USD/EUR). In addition, the dosage form, recommended daily dose according to the manufacturer (µg) were studied, proportion of the recommended daily requirement (Bundesinstitut für Risikobewertung (BfR) 2021a) per dose and testing the product names for suggestiveness with regard to vitamin A as an ingredient. The recommendations on the intake cycle and duration of intake, warnings against overdosing, and information on intake during pregnancy/breastfeeding were also critically examined. Information on possible adverse effects/side effects and interactions was also included. The products were divided into several groups for the analysis:

1. Total preparations (Germany)
2. Monopreparations (Germany)
3. Combination preparations (Germany)
4. Total preparations (USA)
5. Monopreparations (USA)
6. Combination preparations (USA)

The results were then compared and evaluated.

## Results

### Country of manufacture/country of origin of the preparations

**Fig. 2:**
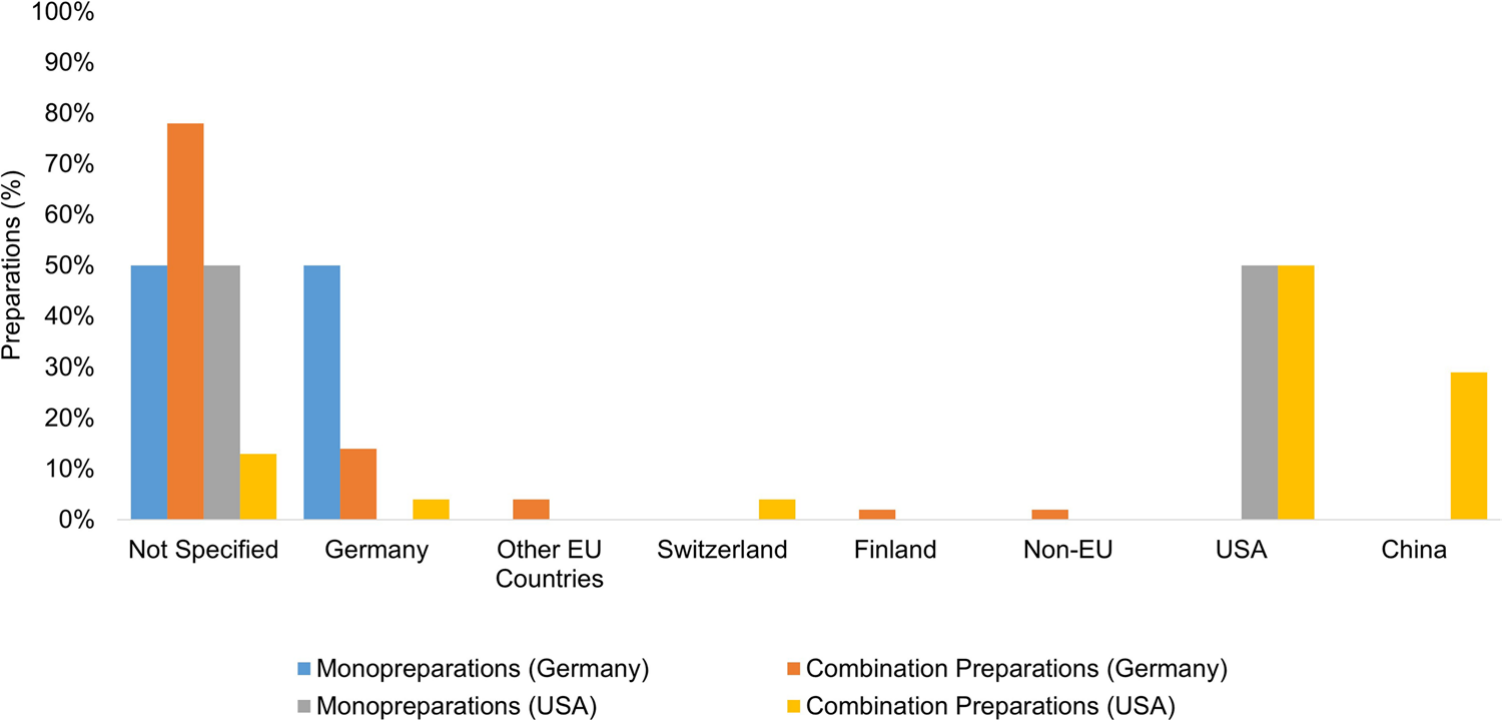
Representation of the countries of manufacture/countries of origin of the individual preparation groups as a bar chart

Figure 2 shows the countries of manufacture/country of origin of all the preparations from Germany (75) and all the preparations from the USA (26). Of the monopreparations available in Germany (26), 50% (13 preparations) state Germany as the country of manufacture/country of origin. The remaining 50% (13 preparations) do not provide any information. For the majority of the combination preparations available in Germany (49), the country of manufacture/country of origin is also not specified (38 preparations; 78%). The combination preparations whose product information contains details are manufactured in descending order of frequency in Germany (7 preparations; 14%), other EU countries (2 preparations; 4%), Finland (1 preparation; 2%), and non-EU countries (1 preparation; 2%). The majority (51 preparations; 68%) of the preparations analyzed that are available in Germany do not indicate the country of manufacture/country of origin. However, dietary supplements fall under food law and are therefore subject to different regulations in both Germany and the USA. According to current EU regulations, it is not sufficient to state the company address for all preparations. The European Parliament’s Food Information Regulation stipulates an obligation to label the origin of a preparation where the consumer must have this information in order to make an informed choice of preparation. In the case of dietary supplements, this information is mandatory if consumers could be misled about the actual country of origin or place of origin of the food supplement without this information (Europäische Kommission 2012). According to an amendment to the Food Information Regulation, since April 1, 2020, the origin of the primary ingredient of a food supplement must also be indicated if it does not correspond to the country of origin or the place of origin of the food (Europäische Kommission 2018). In summary, of the preparations considered that are available in Germany, 13 monopreparations and 38 combination preparations are not sufficiently labeled with regard to their country of manufacture/country of origin. This means that the consumer has no way of knowing where the ingredients of these products come from, what the manufacturing standards are in the countries of production, or how well the ingredients are checked for pesticides and other possible contaminants.

Of the monopreparations available in the USA (2), one is manufactured in the USA (50%), and the other preparation does not specify a country of manufacture/country of origin. Of the combination preparations available in the USA (24), 50% (12 preparations) are manufactured in the USA, then in descending order in China (7 preparations; 29%), Germany (1 preparation; 4%), and Switzerland (1 preparation; 4%). No information was provided on three of the combination preparations (13%). The US preparations examined show their country of manufacture/country of origin more reliably (22 preparations, 85%). In the USA, the FDA requires that the name and place of business of the manufacturer, packer, or distributor be clearly visible on the packaging of all foods and dietary supplements (U.S. Food and Drug Administration 2023a). The country of manufacture/origin is not required. Nevertheless, the country of manufacture/origin is listed for 85% of US products. In summary, US products are more reliably labeled in this regard than products on the German market.

### Dosage form of the preparations

**Fig. 3:**
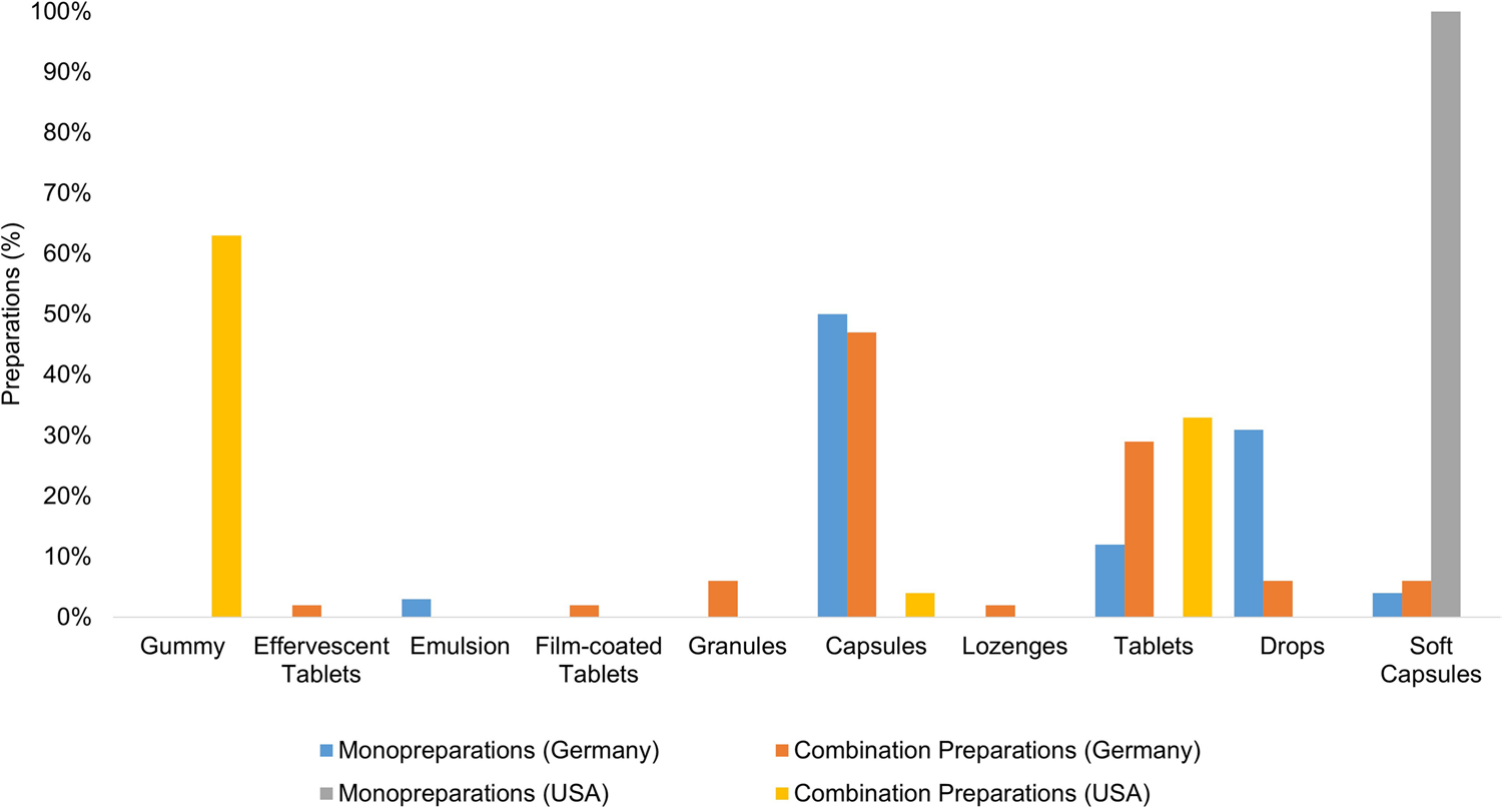
Presentation of the dosage forms of the individual preparation groups as a bar chart

Figure 3 provides an overview of the dosage forms of all the preparations. The monopreparations available in Germany (26) are offered in descending frequency as capsules (13 preparations; 50%), drops (8 preparations; 31%), tablets (3 preparations; 12%), soft capsules (1 preparation; 4%), and emulsion (1 preparation; 4%). The combination preparations available in Germany (49) are in descending frequency as capsules (23 preparations; 47%), tablets (14 preparations; 29%), granules (3 preparations; 6%), drops (3 preparations; 6%), soft capsules (3 preparations; 6%), film-coated tablets (1 preparation; 2%), and lozenges (1 preparation; 2%). The monopreparations available in the USA (2) are offered as soft capsules, while the combination preparations available in the USA (24) are offered in descending frequency as gum drops (15 preparations; 63%), tablets (8 preparations; 33%), and capsules (1 preparation; 4%)**.**

The dosage forms of the preparations differ significantly when looking at the German preparations and the US preparations. Both monopreparations and combination preparations are mainly available in capsule form in Germany, which is only represented by one combination preparation among all the US preparations considered. The US-American combination preparations are mostly offered in the form of gummy drops. This dosage form is not represented in any of the German preparations. If the gummy drops are not stored correctly or handled carelessly, there is a considerable risk that children living in the household will mistake the gummy drops for sweets and accidentally overdose. The second most common form of the preparations in both large groups (all preparations available in Germany and all preparations available in the USA) is in tablet form. Administration as drops is only represented among the German preparations. Here, too, there is a risk of overdosing, as just a few drops of the preparations contain significantly more than the daily intake recommended by the BfR (Bundesinstitut für Risikobewertung (BfR) 2021a). Some already contain almost the UL of vitamin A (3000 µg), and dosing with a pipette is not easy to handle for every consumer.

### Suggestiveness of product names in relation to vitamin A as an ingredient

**Fig. 4:**
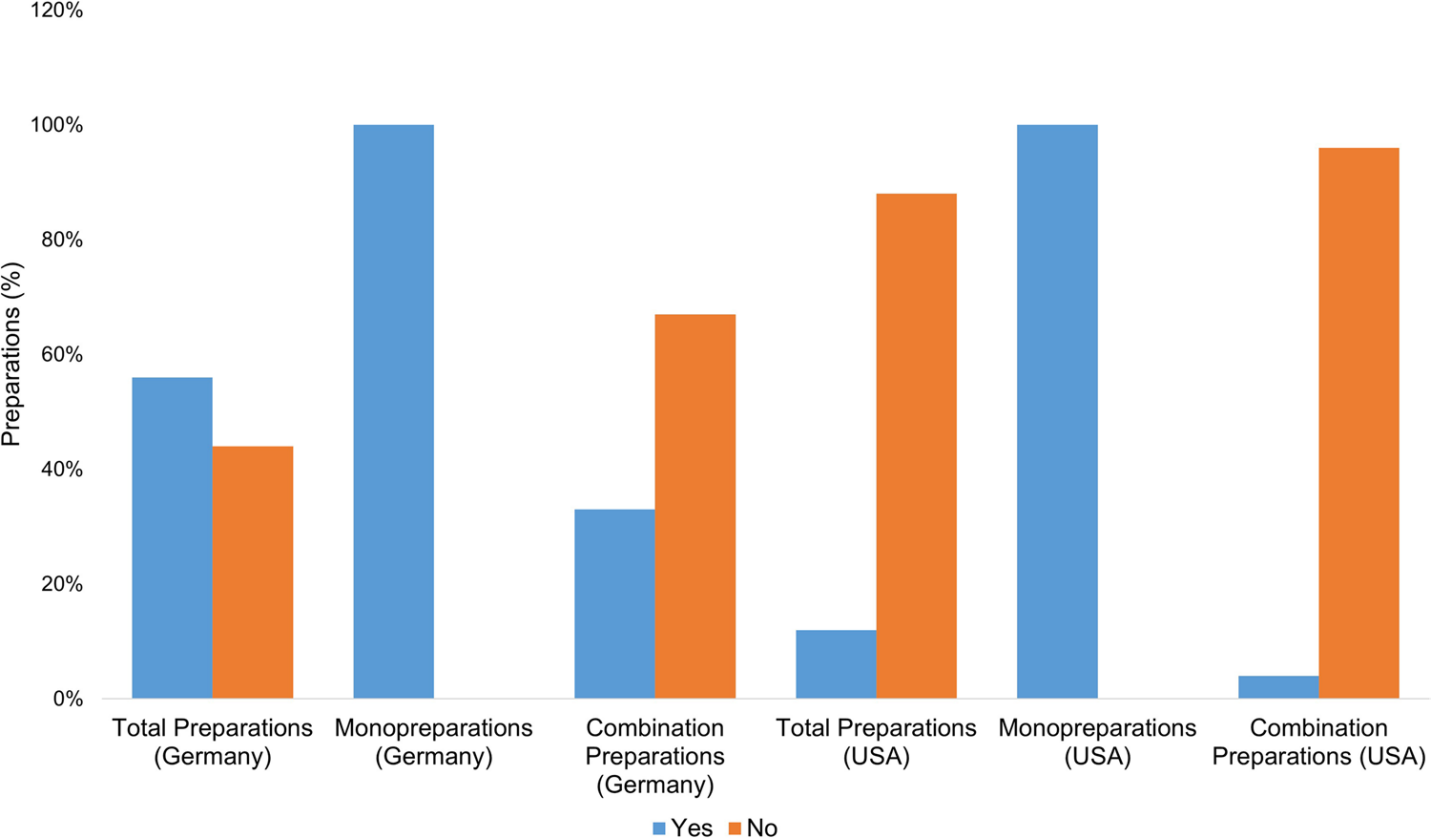
Representation of the suggestiveness of the product names for vitamin A as an ingredient in the form of a bar chart, whereby the proportion of preparations with suggestive names is colored blue, and the proportion of preparations with non-suggestive names is colored orange

Figure 4 shows how often the product names are suggestive of the ingredient vitamin A. In evaluating the product names, the mention of vitamin A was considered positively suggestive. Names such as “multivitamin” were assessed as “non-suggestive,” as consumers cannot be expected to infer that vitamin A is an ingredient.

Of all the preparations available in Germany (75), the names of 42 preparations (56%) are suggestive, while the names of 33 preparations (44%) are not suggestive. The monopreparations available in Germany all have suggestive product names; of the combination preparations, 16 (32%) have a suggestive product name, and 33 (67%) do not. Of all the preparations available in the USA (26), three product names (12%) are suggestive; 23 product names (88%) are not. Both monopreparations available in the USA (2; 100%) have suggestive product names. The combination preparations available in the USA (24) do not have a suggestive name in 23 cases (96%). One mixed preparation (4%) has a suggestive name. All of the German vitamin A monopreparations have a suggestive product name, as do the US monopreparations. In the group of German preparations, 67% (33 preparations) of the combination preparations cannot be reliably identified as containing vitamin A by consumers. In the group of US-American combination preparations, 96% (23 preparations) of the product names are not suggestive and therefore even more difficult for consumers to identify as containing vitamin A in their entirety. If consumers take several supplements without knowing the vitamin A content, this represents a considerable risk factor for an overdose.

### Daily therapy costs (DTC) (€)

**Fig. 5:**
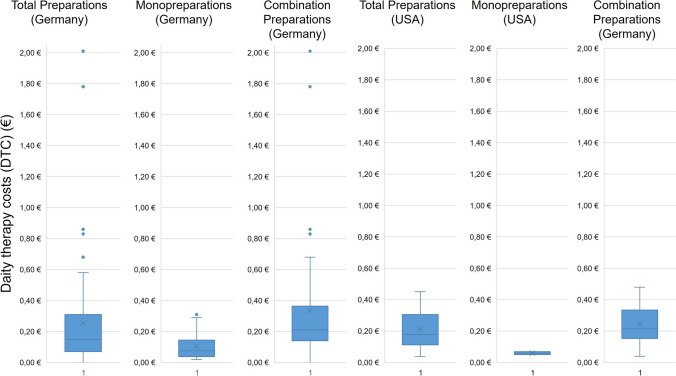
Representation of the daily therapy costs (DTC) (€) of all preparations as a box chart. The box corresponds to the middle 50% of the data. It is delimited by the upper and lower quartiles. The lines in the box indicate the median. The crosses mark the mean values. The upper whiskers mark the maximum values, and the lower whiskers mark the minimum values. The dots outside the boxes highlight the outliers

Figure 5 shows the daily therapy costs of the preparations under consideration as a box chart. For all preparations available in Germany, the median is € 0.15, and the mean is € 0.26. The minimum value is € 0.02, and the maximum is € 2.01. Looking at the monopreparations and combination preparations available in Germany separately, the daily treatment costs for combination preparations are higher, with a median of € 0.22 and a mean value of € 0.34 compared to the monopreparations with a median of € 0.08 and a mean value of € 0.10. In addition, there are outliers among the combination preparations which, with daily therapy costs of € 1.78 and € 2.01, are significantly above the median and also above the mean of both groups.

For all preparations available in the USA, the median daily treatment cost is € 0.18, and the mean is € 0.22. The minimum value is € 0.04. The minimum value is € 0.04, and the maximum value is € 0.45. The median and mean value of the monopreparations is € 0.06. The combination preparations are also more expensive here compared to the monopreparations, with a median of € 0.20 and mean value of € 0.23. There are no outliers among the US preparations.

As dietary supplements are not subject to pharmaceutical regulations and therefore not subject to the German Drug Price Ordinance (AMPreisV 2023), a broad price spectrum is evident here. The German monopreparations (median € 0.08) are almost on a par with the US monopreparations (median € 0.06), although it should be noted that the US monopreparations are only represented by two preparations. With a median of € 0.22, the German combination preparations have the highest daily therapy costs and at the same time, the widest range within the groups considered. In addition, several high-priced outliers with DTC of up to € 2.01 are represented. Compared to this, the DTC of the US combination preparations are also somewhat lower overall, with a median of € 0.20. There are no outliers among the US preparations. Thus, the German market is less well controlled than the US market.

### Amount of the recommended daily dose of vitamin A (µg) according to the manufacturer

**Fig. 6:**
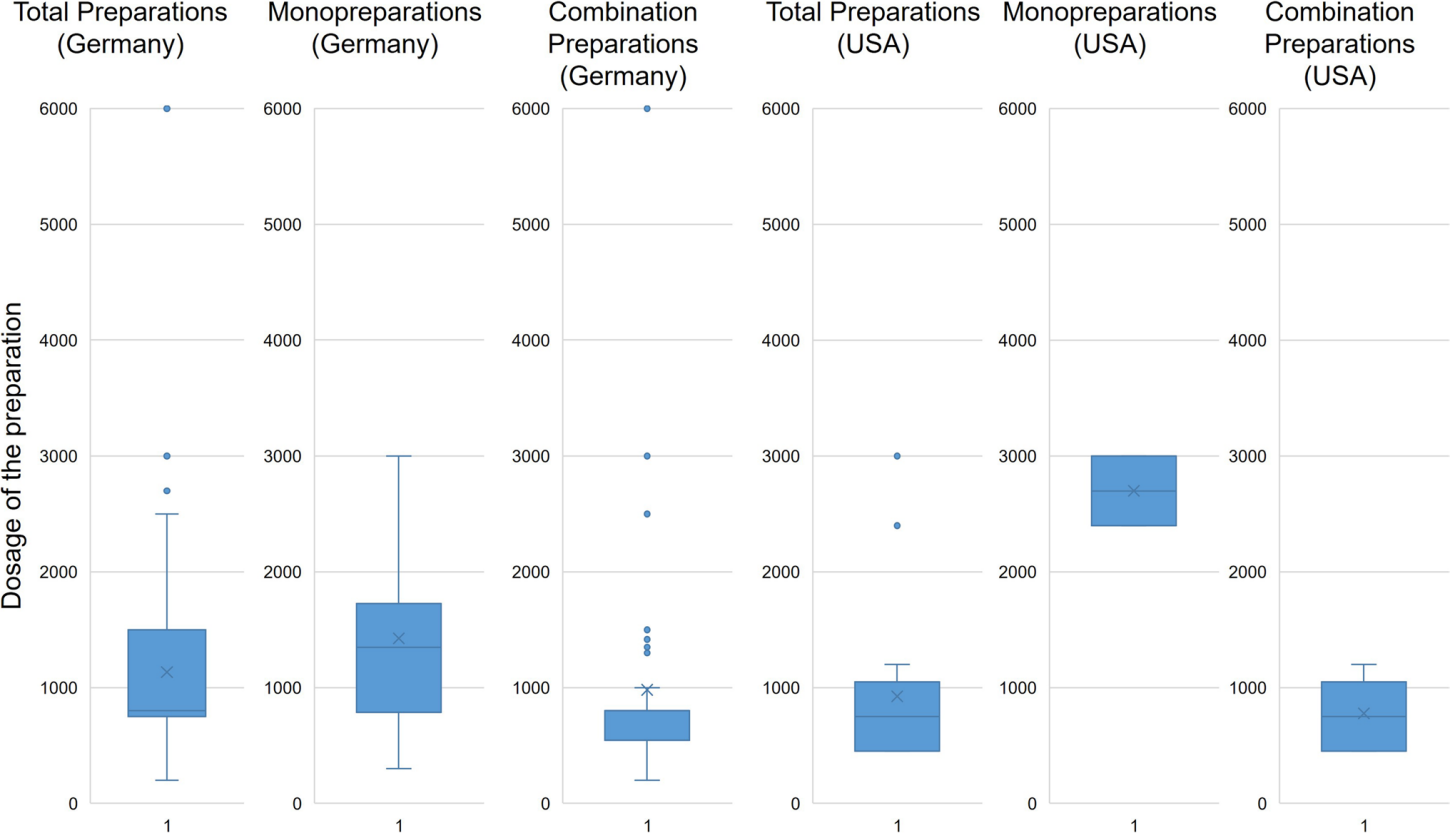
Representation of the daily dose of vitamin A (µg) recommended by the manufacturer for all preparations as a box chart. The box corresponds to the middle 50% of the data. It is delimited by the upper and lower quartiles. The lines in the box indicate the median. The crosses mark the mean values. The upper whiskers mark the maximum values, and the lower whiskers mark the minimum values. The dots outside the boxes highlight the outliers

Figure 6 shows the daily dose of vitamin A recommended by the manufacturer for all preparations. For all preparations available in Germany, a median of 800 µg and a mean of 1135 µg of vitamin A are recommended as a daily dose. Looking at the mono- and combination preparations, the median is 1350 µg and 800 µg, respectively, and the mean is 1425 µg and 980 µg, respectively. The minimum value for the monopreparations is 300 µg and for the combination preparations 200 µg. The maximum value in the group of monopreparations is 3000 µg and in the group of combination preparations 6000 µg. The maximum value of the combination preparations is an outlier. These maximum values are the UL and twice the UL. Further graphical representations of the present results are found in Figures S1, S2, and S3 of the supplementary figures. Whereby Figure S1 shows the frequency of a daily dose equal to or below the recommended daily intake of the German preparations, Figure S2 shows the frequency of a daily dose above the recommended daily intake of the German preparations, and Figure S3 shows the frequency of a daily dose equal to or above the UL of the German preparations, each as a bar chart.

For all US preparations, the median is 750 µg, and the mean is 926 µg. The median and mean values are significantly higher for the monopreparations at 2700 µg each than for the combination preparations at 750 µg and 778 µg. However, only two preparations are represented in the group of US monopreparations, which limits comparability. The minimum values of the mono- and combination preparations are 2400 µg and 450 µg, and the maximum values are 3000 µg and 1200 µg. Additional graphical representations of the present results are shown in Figures S4, S5, and S6 of the supplementary figures. Whereby Figure S4 shows the frequency of a daily dose equal to or below the recommended daily intake of the US preparations, Figure S5 shows the frequency of a daily dose above the recommended daily intake of the US preparations, and Figure S6 shows the frequency of a daily dose equal to or above the UL of the US preparations, each as a bar chart. Sixty-seven percent (50 preparations) of the German vitamin A preparations (75) contain exactly or less than the recommended daily intake. A quarter of the German preparations (19 preparations, 25%) contain more than the recommended daily intake, and 8% (six preparations) contain exactly the UL or even more than the UL recommended for vitamin A. The preparation (mixed preparation) with the highest dosage (6000 µg per dose/day) corresponds to twice the UL, 7.7 times the recommended daily intake of vitamin A, and 15 times the recommended amount per daily dose in dietary supplements according to BfR (Bundesinstitut für Risikobewertung (BfR) 2021a). With regard to all US preparations, the manufacturer’s recommended daily dose for 14 preparations (54%) is below the recommended daily dose. In 12 preparations (46%), the recommended daily dose is exactly at or above the recommended intake. One preparation (4%) contains exactly the UL per daily dose. Thus, again, the German market is less well controlled than the US market.

This shows that the dosage recommendations often already exceed the recommended daily intake of vitamin A and even more clearly the daily dose of 400 µg for vitamin A recommended by the BfR for dietary supplements. In some cases, the combination preparations contain more vitamin A than the monopreparations, which can result in an accidental overdose and pose a risk to the consumer. Especially as vitamin A is also taken in with food, which is not yet taken into account when considering and evaluating the dosages.

Various studies show that the recommended daily intake of vitamin A in the Federal Republic of Germany (A. Domke et al. 2008; Adolf et al. 1995; Kersting et al. 2004) and in the USA (World Health Organization 2009, Zhao et al. 2022) is largely achieved, and no additional intake of vitamin A is usually necessary. In this context, the BfR recommends avoiding the intake of additional vitamin A via dietary supplements wherever possible. However, if supplementation does take place, a maximum daily dose of 400 µg is recommended (Bundesinstitut für Risikobewertung (BfR) 2021a). The FDA makes no recommendations on this.

Long-term use of high-dose supplements poses significant health risks such as liver cell damage (Kowalski et al. 1994; Nollevaux et al. 2006; Stickel et al. 2011) or teratogenic effects in pregnant women. These can already occur with a daily intake of over 3000 µg over a longer period of time. In a retrospective study involving 22,748 pregnant women, women who consumed more than 3000 µg of vitamin A daily in the form of dietary supplements during pregnancy, especially in the first trimester showed a 4.8-fold higher risk of giving birth to a child with a neural tube defect than women who consumed 1500 µg of vitamin A daily in the form of dietary supplements (Rothman et al. 1995). Further studies show contradictory results with regard to the threshold dose, so that no threshold dose has yet been defined (Hayes et al. 1981; Hendrickx et al. 2000). Following an expert assessment by the European Commission, the UL for vitamin A for pregnant women was set at 3000 µg per day (SCCS 2016, SCF 2002). This UL was also set by the Institute of Medicine for the USA (FNB/IOM 2002). In addition, there are very high tolerance values for the ingredients of dietary supplements. In Germany, a vitamin A supplement may contain 50% more or 20% less vitamin A than stated in the product information (Europäische Kommission 2012). This provides scope for additional inaccuracies in the dosage. For example, a preparation that states a quantity of 3000 µg per daily dose would still be within the legal limits with a real quantity of 4500 µg. However, this dosage is already well above the UL, which poses a risk to consumers. In the USA, the actual amount of the ingredient in Class I foods, these are foods fortified or manufactured with nutrients according to the FDA’s CFR, must be at least equal to the amount stated on the label (U.S. Food and Drug Administration 2023c). A shortfall in the amount is therefore not legally tolerated. Exceeding the amount stated on the label is “acceptable under current good manufacturing practice” (U.S. Food and Drug Administration 2023d). An exact definition of the permitted deviation in percent from the declared value cannot be found in the regulation. This means that in the USA, a dose lower than the declared value is not permitted, but exceeding the amount stated on the label is acceptable to a reasonable extent. Without a definition of the maximum tolerated exceedance of the value, there are also risks for consumers.

### User-friendly indication of the preparation dose (%) of the BfR’s recommended daily dose

**Fig. 7:**
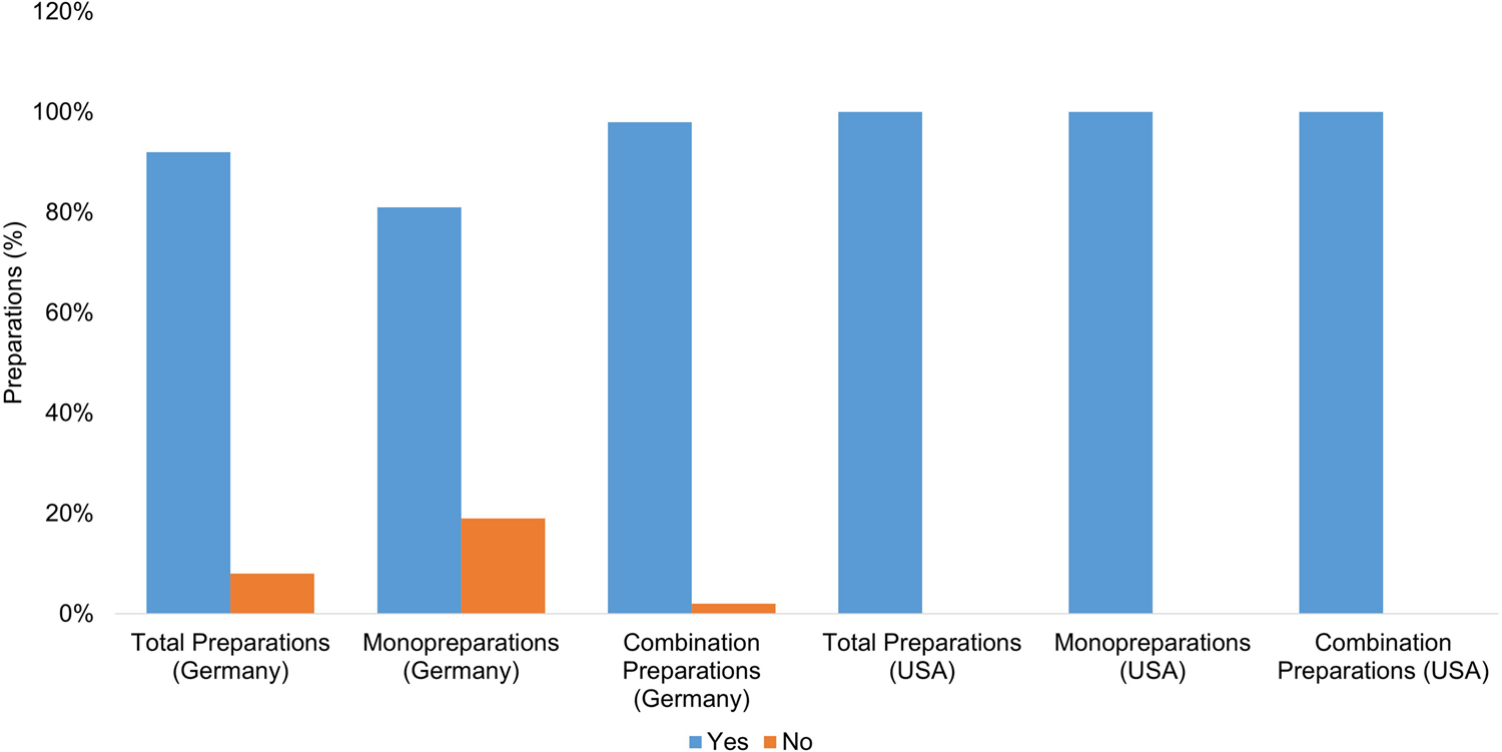
Representation of the frequency of user-friendliness of the dosage information of the preparations as a bar chart, whereby the proportion of preparations with user-friendly dosage information is colored blue, and the proportion of preparations without is colored orange

Figure 7 shows the user-friendliness of the dosage information for the preparations. This refers to the indication that the percentage of the daily recommended intake (Bundesinstitut für Risikobewertung (BfR) 2021a) is covered by the recommended daily dose of the preparation. Of all the preparations available in Germany, six (8%) do not provide user-friendly information on the dose as a percentage of the recommended daily dose. The preparations are a mixed preparation with a recommended daily dose of 400 µg vitamin A and five monopreparations with daily doses of 750 µg, 1500 µg (3 ×), and 2400 µg. All of the preparations considered, which are available in the USA, contain a user-friendly indication of the dose in % of the recommended daily dose.

Although it is mandatory in Germany to state the vitamin A intake per daily dose as a percentage of the defined reference amount for dietary supplements containing vitamin A (Europäische Kommission 2012), some preparations are inadequately labeled. The lack of user-friendly information in percentage can encourage overdosing, as very few consumers are familiar with the reference amount of vitamin A. In the USA, it is also mandatory to label dietary supplements with the amount of vitamin A they contain as a percentage of the daily reference amount (U.S. Food and Drug Administration 2023f). Another time, the market in Germany is less well controlled than in the USA.

### Overdose warning for the preparations

**Fig. 8:**
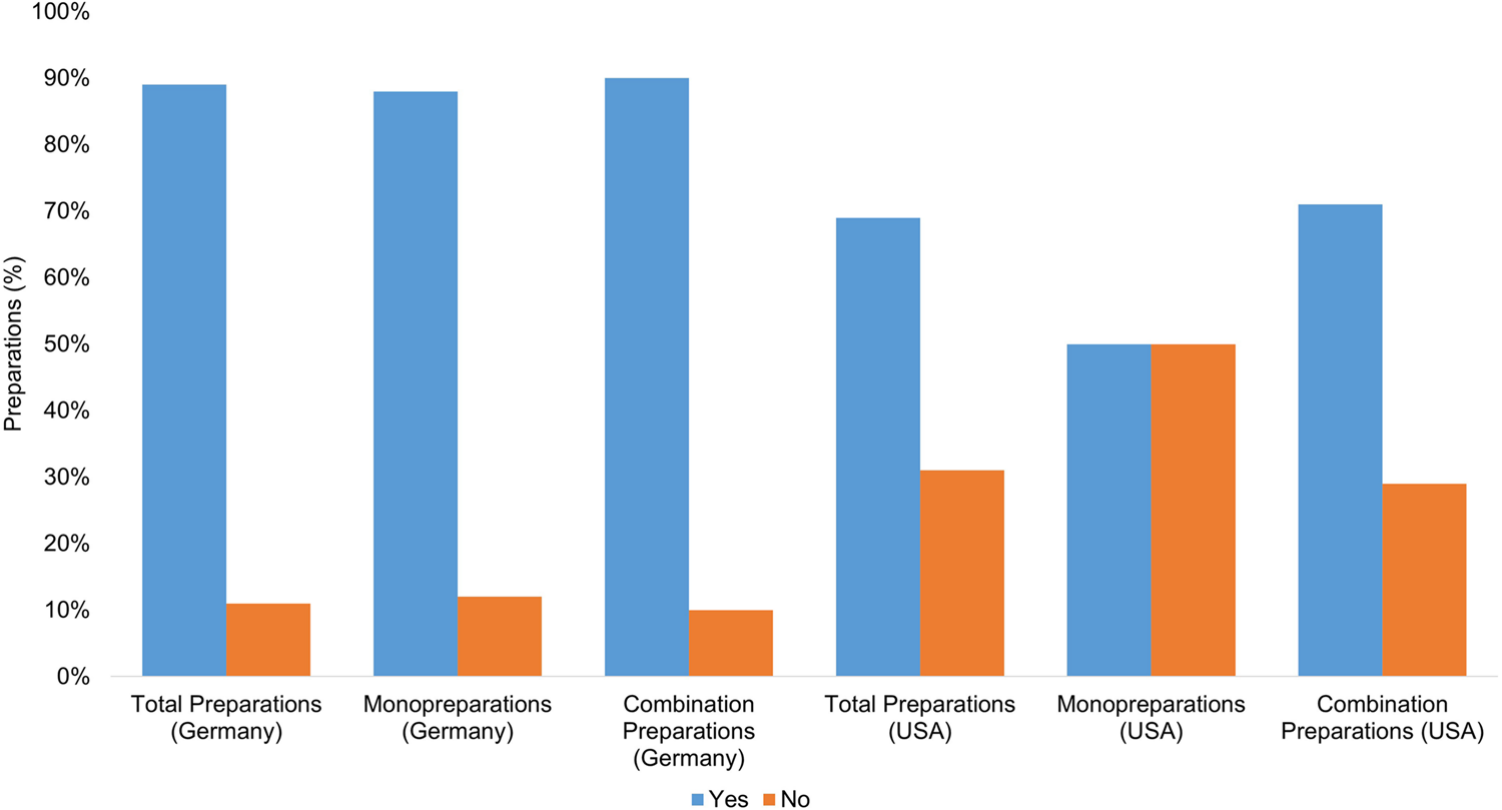
Representation of the frequency of a warning before overdose of the preparations as a bar chart, whereby the proportion of preparations with a warning before overdose is colored blue, and the proportion of preparations without a warning is colored orange

Figure 8 shows in how many cases there is a warning against overdose in the product information or, if present, on the outer packaging. The following wording was assumed for a positive evaluation: “The recommended daily intake should not be exceeded.”

Of all the preparations available in Germany (75), 67 preparations (89%) carry a warning, while 8 preparations (11%) do not.

Of the total number of US preparations examined (26), 18 (69%) carry an overdose warning, and eight preparations (31%) do not. The obligation to label dietary supplements with a warning against overdose is regulated by law in Germany at EU level. All dietary supplements sold in the EU must carry the warning “Do not exceed the recommended daily intake” (Europäische Kommission (2012)). The majority of German products (67 products; 89%) comply with this obligation. The poorly labeled preparations (8 preparations; 11%) predominantly already contain the recommended daily dose of vitamin A. Two preparations even contain around twice and three times the recommended daily dose or four and six times the recommended daily dose for dietary supplements. This warning is not mandatory in the USA (U.S. Food and Drug Administration (2023e)). Almost a third of the preparations (8 preparations; 31%) do not carry a warning. Here too, the dosages of the preparations concerned are sometimes higher than the recommended daily dose. The preparations with no warning were one monopreparation (50% of the monopreparations) with a daily dose of 2400 µg and seven combination preparations (29% of the combination preparations) with doses of 450 µg to a maximum of 1200 µg. The absence of this warning represents a further risk for consumers.

### Warnings on taking the preparations during pregnancy and breastfeeding

**Fig. 9:**
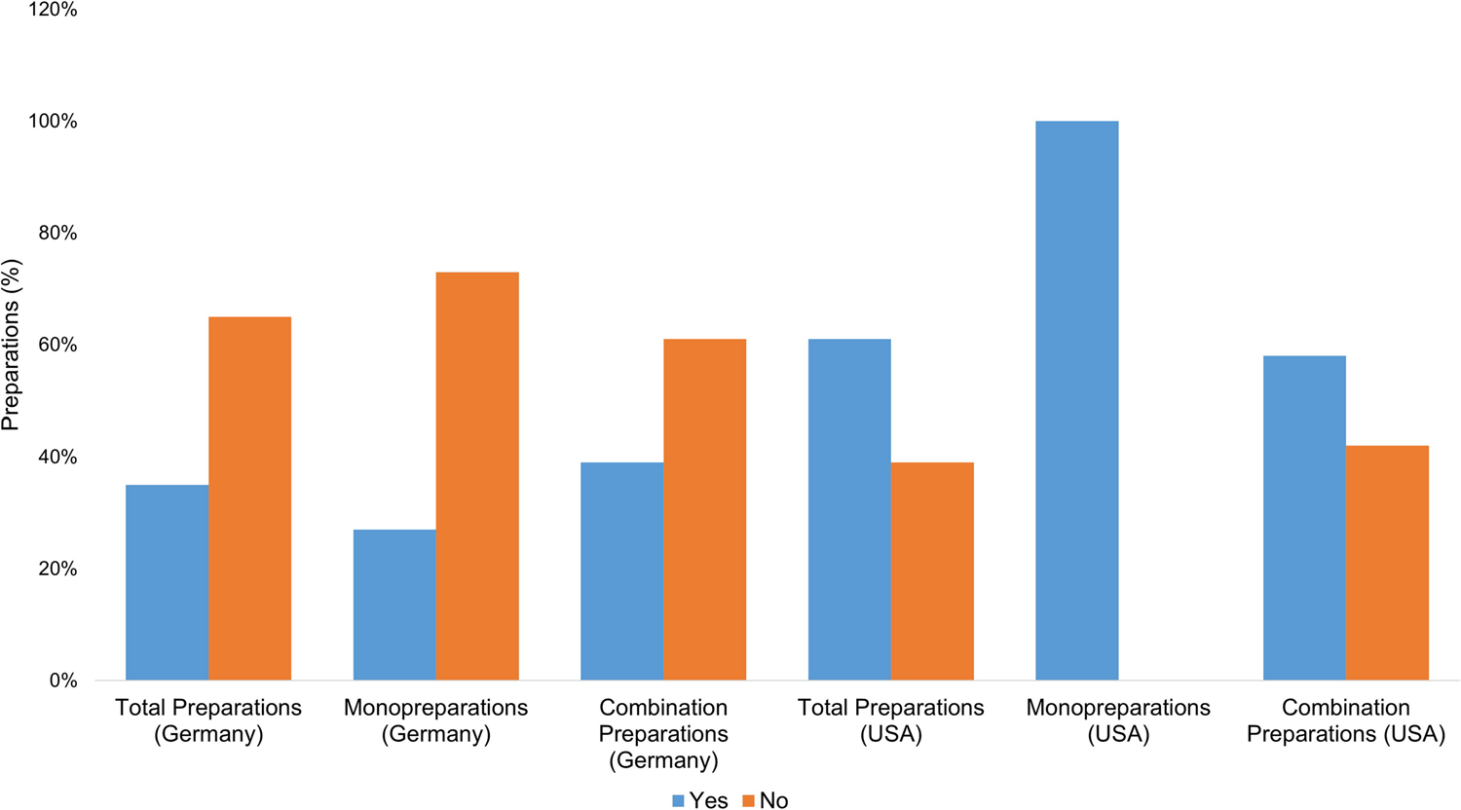
Representation of the frequency of a warning before taking the preparations during pregnancy and breastfeeding in the form of a bar chart, whereby the proportion of preparations with a warning is colored blue, and the proportion of preparations without a warning is colored orange

Figure 9 demonstrates the presence of a warning regarding the use of the preparations during pregnancy and breastfeeding in the product information or, if present, on the outer packaging. Looking at all of the preparations available in Germany (75), the majority (49 preparations; 65%) do not carry a warning against use during pregnancy and breastfeeding. The preparations without a warning are 19 monopreparations (73% of the monopreparations) with daily dosages of 300 µg to a maximum of 3000 µg, whereby the maximum value already corresponds to the UL. Among the combination preparations (49), 30 preparations (61%) with daily doses of 200 µg to a maximum of 6000 µg also carry no warning, with the maximum value corresponding to twice the UL.

Of the US preparations considered (26), a total of 10 preparations (39%) are free of the warning. The preparations are exclusively combination preparations (42% of the combination preparations) with dosages of 450 µg to a maximum of 1200 µg per daily dose. The warnings for taking vitamin A-containing dietary supplements during pregnancy and breastfeeding are regulated by law in Germany and the USA (NemV 2004, U.S. Food and Drug Administration (2023g)). In both countries, there is an obligation to label dietary supplements that are not safe for pregnant and breastfeeding women with a warning. Vitamin A, as a potentially teratogenic substance, is subject to this labeling requirement. Only around a quarter of German products (26 products; 25%) fulfill this obligation, while two-thirds of US products (16 products; 16%) do so. Again, the German market is less well regulated than the market in the USA. This aspect must be viewed very critically in the case of high-dose preparations, as the teratogenic effect of vitamin A has been well established in retrospective studies and also in animal experiments, although no exact threshold dose has been defined (Hayes et al. 1981; Rothman et al. 1995). A retrospective study (Rothman et al. 1995) found that taking more than 3000 µg of vitamin A over an extended period, particularly during the first trimester, increases the risk of fetal malformation. In the study in question, the safe dose is therefore set at a maximum of 3000 µg per day. Other studies have set the safe dose of vitamin A much higher, such as 25,000 to 37,000 IU/day, which is 7500–10,500 µg/day (Hendrickx et al. 2000). However, it is certain that an overdose of vitamin A over a long period of time increases the risk of fetal malformation. The threshold dose above which there is an increased risk of malformation cannot yet be determined with certainty based on the current data. Pregnant and breastfeeding women are a particularly vulnerable group of consumers. They should be adequately protected by proper labeling and safe dosage.

### Recommendation for taking the preparations during pregnancy and breastfeeding

**Fig. 10:**
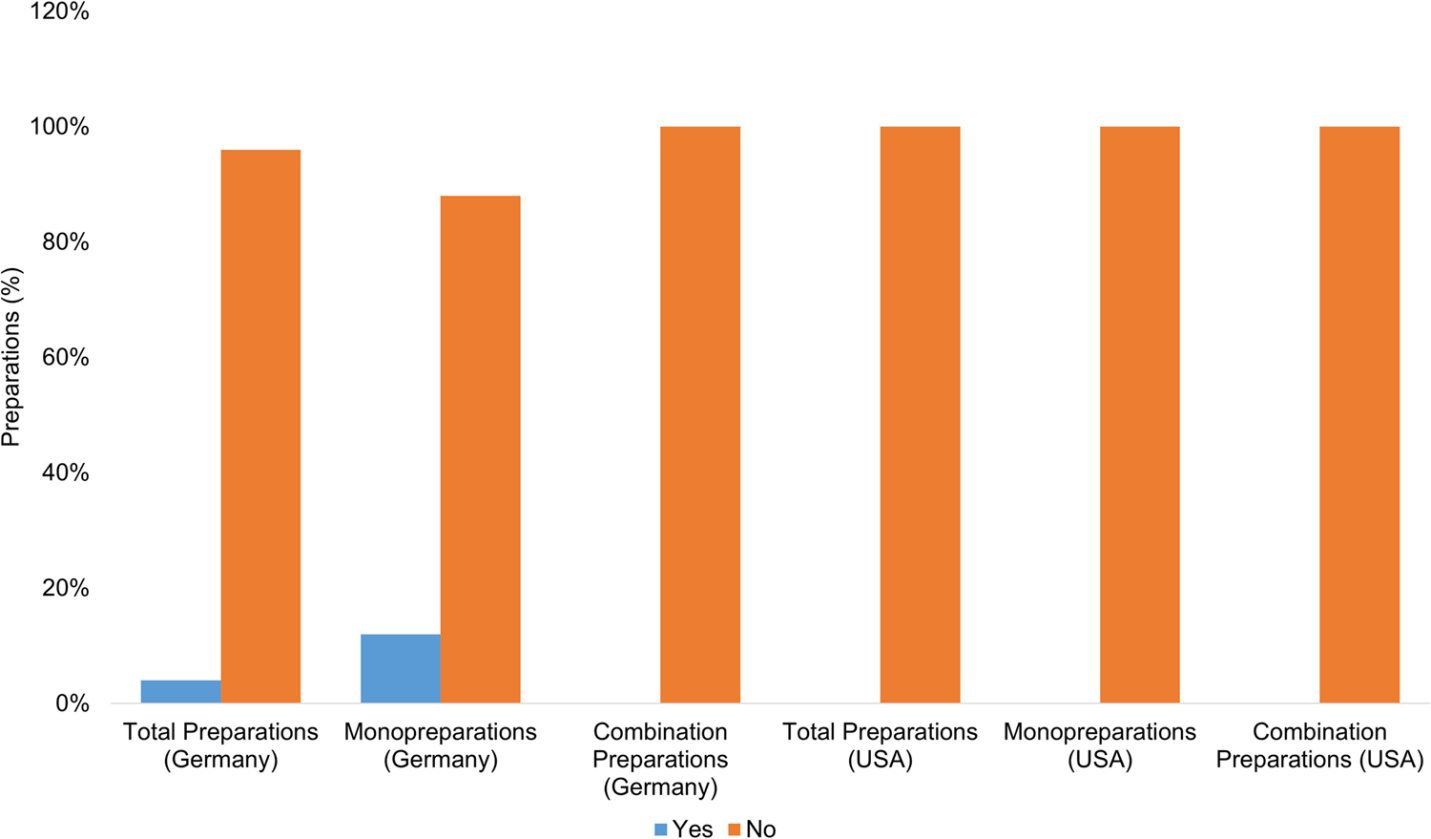
Representation of the frequency of a recommendation to take the preparations during pregnancy and breastfeeding in the form of a bar chart, whereby the proportion of preparations with a recommendation is colored blue, and the proportion of preparations without a recommendation is colored orange

Figure 10 illustrates how many preparations are recommended for use during pregnancy and breastfeeding. Three monopreparations available in Germany (12% of the monopreparations) recommend taking them during pregnancy and breastfeeding in the product information. The preparations in question contain from 800 µg to a maximum of 3000 µg vitamin A per daily dose. The maximum value already corresponds to the UL. The preparation with a daily dosage of 800 µg also contains no warning against overdosing.

The analysis of the US preparations showed that none of the preparations recommends taking them during pregnancy and breastfeeding. The analysis of the US preparations showed that none of the preparations recommended use during pregnancy and breastfeeding. Other preparations recommend their use during pregnancy and lactation, despite the risks already mentioned. The preparations (3%) are three monopreparations available in Germany. These products contain between 800 µg and a maximum of 3000 µg of vitamin A per daily dose. The maximum level already corresponds to the UL. Based on the facts presented, pregnant and breastfeeding women should not be recommended to take vitamin A supplements without a proven vitamin A deficiency, medical consultation, and individual advice.

### Information on possible undesirable effects/side effects

In the assessment of the preparations with regard to information on possible undesirable effects/side effects, there is only one indication for a monopreparation available in Germany with a daily dose of 3000 µg. Here, it is first pointed out that there is a possibility of overdosage. Headaches, dizziness, nausea, and hair loss are mentioned as possible adverse effects/side effects.

In this context, there was no information on possible adverse effects/side effects for any of the US preparations examined. The graphical representation of the present results is found in Figure S7.

### Possible interactions with medicines

None of the preparations considered that are available in Germany or the USA addresses possible interactions with medicinal products. As vitamin A is not a medicinal product and is subject to food regulations, the disclosure of possible interactions with medicinal products is not mandatory.

### Naming explicit target groups for the preparations

The target groups in the product name and product information were examined for all preparations. None of the preparations considered that are available in Germany mentions an explicit target group; the product information is only aimed neutrally at adults.

The US preparations mostly specify a target group through the names of the preparations. Of the total number of preparations (26), 12 (46%) are recommended for women, 7 (27%) for men, and 7 (27%) are advertised neutrally (for adults) in the product information. The corresponding graphical representation of the present results is found in Figure S8.

The naming of different target groups makes sense if the dosage of the preparation takes into account the recommended daily dose for this target group. People with an increased risk of osteoporosis should not take high-dose vitamin A supplements over a longer period of time, for example, due to a possible negative impact on bone health (Green et al. 2017; Lind et al. 2017; Yorgan et al. 2016). The UL for this consumer group is 1500 µg vitamin A (SCF 2002). Pregnant women also represent a special consumer group, as already discussed. The recommended daily doses of the preparations vary greatly, irrespective of the target group mentioned. It appears that the naming of these target groups does not result from an individual perception of the groups, but merely serves marketing purposes.

### Recommendations on the duration of use

Two of the 75 preparations available in Germany give a recommendation on the duration of use, although this is not precisely defined.

The two combination preparations, each with a daily dose of 800 µg, state the following: “use over 1–2 months several times a year is recommended” and “a break is recommended after 10 months of use.” The recommendations are not further specified.

None of the US preparations under consideration gives a recommendation on the duration of use.

## Discussion

### Country of manufacture/country of origin of the preparations

US products are more reliably labeled in this regard than products on the German market. The indication of the country of manufacture or origin of dietary supplements is crucial for consumers as it provides important information about the quality and safety of a product. Accurate labeling enables consumers to make informed choices and to be aware of, and minimize, potential risks. Labeling is mandatory in Germany, but not in the USA. In theory, this results in more uncertainties and risks for US consumers. Naming the country of manufacture can indicate whether a product was manufactured in a country with strict quality standards and regulations. Consumers can avoid products from countries with known food safety or quality issues in favor of products from countries with stricter controls. In addition, clear and accurate labeling creates consumer confidence and promotes transparency in the dietary supplement industry. Consumers should always have the right to know where their dietary supplements come from and under what conditions they were produced. With regard to the control of claims, stricter monitoring and enforcement of labeling regulations in Germany and the introduction of clear legislation in the USA are desirable and necessary. Government authorities and regulators need to ensure that manufacturers provide correct and accurate origin claims and that these claims are verifiable. In addition, mandatory certification or quality standards for producers would be useful to ensure that products meet the highest quality and safety standards. This would not only increase consumer confidence but would also help to protect consumer health.

### Dosage form of the preparations

There are advantages and disadvantages to selecting a specific dosage form. Gummy drops are favored for their taste and ease of ingestion, but they present a danger of children confusing them with candy and consuming an excessive amount. Capsules, soft capsules, and tablets are simple to dose and have a longer shelf life, but their size may make them difficult to swallow. Overall, drops allow for quick absorption and more flexible dosing, but they can also pose risks for overdosing. Additionally, drops have an unpleasant taste and a shorter shelf life. Ultimately, the choice of dosage form depends on individual preferences, needs, and cultural preferences. It is important to weigh the pros and cons of each dosage form to select the most suitable option according to individual needs.

### Suggestiveness of product names in relation to vitamin A as an ingredient

In Germany, approximately 67% of combination preparations and in the USA, as many as 96% of combination products do not clearly indicate the vitamin A content. This lack of clarity makes it considerably more difficult for consumers to identify dietary supplements containing vitamin A. To better protect consumers, reforms to the labeling guidelines for dietary supplements are needed to ensure that product names clearly and unambiguously indicate the vitamin A content. Regulators should implement stricter controls to ensure that labeling is clear and not misleading. Public education campaigns could educate consumers about the risks of vitamin A and vitamin overdoses in general and encourage them to research supplements before taking them and discuss the need for supplementation with their physician. Increased cooperation between regulators, manufacturers, and consumers is necessary to ensure the safest possible use of dietary supplements.

### Daily therapy costs (DTC) (€)

The discussion about the necessity and appropriateness of price regulation or price control of dietary supplements in Germany and the USA is complex, as there are many aspects to consider. Dietary supplements can play an important role in nutrient supplementation if they are properly indicated for use. This is especially true for people with specific dietary needs or restrictions. Price regulation or caps could help more consumers access high-quality, safety-tested products and protect financially vulnerable consumers from purchasing inferior or potentially harmful products. This would protect consumer health. However, the economic aspects must also be taken into account. Price regulation could have a negative impact on small companies and start-ups. They may not be able to bear the cost of complying with price regulations in competition with large companies, which could lead to reduced competition. Companies could also stop producing dietary supplements due to the lower profit margin, thereby reducing supply. In addition, price regulations could lead to the relocation of the production of dietary supplements to countries where production is cheaper but quality and safety standards are lower. This could have a negative impact on quality and therefore on consumer protection. In view of these aspects, the issue of price regulation of dietary supplements requires careful consideration of the different interests of consumers and companies, taking into account both market and economic dynamics as well as consumer safety.

### Amount of the recommended daily dose of vitamin A (µg) according to the manufacturer

Due to the significant increase in the intake of dietary supplements by the population, a discussion and rethinking of the regulation of the dosage of vitamin A and fat-soluble vitamins in dietary supplements in general is overdue. The establishment of mandatory maximum levels and tighter tolerances in the event of deviations is essential to minimize potential health risks. Subsequently, monitoring and enforcement of these requirements would be crucial to ensure that manufacturers do not exceed the limits and place potentially harmful products on the market. This would require effective monitoring of the dietary supplement industry and appropriate legal action against violators. It would be important to involve toxicology experts in the process of developing regulatory requirements. This would ensure that regulations are scientifically sound, practicable, and in the best interests of consumers. It would also be important to raise consumer awareness of the risks of overdose and to educate consumers on the safe use of dietary supplements. Comprehensive education could help consumers make the right choices about supplementation and avoid potential dangers. The development of international standards would also be desirable to ensure safe regulation and effective protection against cross-border trade in unregulated and unmonitored products. Close transnational cooperation could ensure that the same standards are followed worldwide. Again, it should be noted that only close cooperation between government authorities, manufacturers, and consumers can ensure the safe use of dietary supplements and minimize potential health risks.

### User-friendly indication of the preparation dose (%) of the BfR’s recommended daily dose

A user-friendly declaration of the vitamin A content of dietary supplements as a percentage of the recommended daily allowance provides consumers with important information about the contribution of the food supplement to the daily intake of vitamin A. This transparency not only promotes consumer understanding but also contributes to safety and risk minimization by helping to avoid potential overdoses. Consistent labeling makes it possible to compare different products and helps to improve consumer information, safety, and transparency. However, in order for consumers to properly evaluate the claim, they need to know the recommended daily intake and approximate dietary intake of vitamin A. Again, extensive public education is essential for the safe use of over-the-counter dietary supplements.

### Overdose warning for the preparations

Overall, the creation of regulatory requirements could help establish uniform labeling and warning requirements for vitamin A dietary supplements and dietary supplements in general. This would require the establishment of uniform labeling requirements that clearly define the form of overdose warnings. The goal should be to establish uniform regulations in all countries. Effective monitoring and enforcement of these regulations would then be essential. Regulatory requirements should be scientifically sound, workable, and in the best interests of consumers. This would help to protect the safety and health of consumers and also allow for cross-border distribution under the applicable regulations.

### Warnings on taking the preparations during pregnancy and breastfeeding

The “Not suitable for Pregnant or Nursing Women” label is essential to protect pregnant women from the potential risks of excessive vitamin A intake. This warning should be clearly and prominently displayed on the package. Overall, it is extremely important that pregnant women do not take vitamin A supplements unless a safe deficiency has been previously diagnosed by a physician. Clear labeling of such products as “not for use by pregnant or breastfeeding women” will help protect the health of mother and child and minimize potential risks.

### Recommendation for taking the preparations during pregnancy and breastfeeding

With reference to the discussions in the previous points, we strongly advise against recommending the use of vitamin A supplements during pregnancy and breastfeeding in the absence of a documented vitamin A deficiency.

### Information on possible undesirable effects/side effects

Of all the preparations considered, only one contains a warning about possible side effects and possible overdose. This preparation, which is available in Germany, indicates the symptoms that may occur in the event of an overdose. As some preparations are very highly dosed, in some cases reaching or exceeding the UL of 3000 µg, the risk of a symptomatic overdose is significantly increased. It is therefore advisable to inform consumers about the possible symptoms of an overdose.

### Possible interactions with medicines

None of the product information mentions possible interactions between vitamin A and medicines. However, there is a potential for interaction. For example, if a consumer is already taking tretinoin (a vitamin A derivative), there is a considerable potential for overdosing if a vitamin A preparation is taken at the same time. High doses of vitamin A can also reduce the absorption of vitamin K in the intestine and therefore lead to an increased risk of bleeding if vitamin K antagonists such as phenproucumon are taken at the same time (Biesalski 2016). Medical advice for consumers with existing medication before taking vitamin A supplements would be useful and should be included as a recommendation in the product information. Consideration should be given to making vitamin A supplements available only in pharmacies so that expert advice can be sought before use.

### Naming explicit target groups for the preparations

Clear labeling of dietary supplements according to their target groups allows consumers to better select products that meet their specific needs. In addition, labeling can help minimize potential risks. Clear target group identification can also help increase manufacturer accountability and promote compliance with applicable regulations. By clearly communicating the specific target groups for their products, manufacturers can help avoid misunderstandings and ensure that their products are used properly. Overall, therefore, labeling of dietary supplements can help to improve consumer information, increase safety of use, and promote regulatory compliance. Target group labeling is only useful if the dosage of the product takes into account the recommended daily dose for that target group.

### Recommendations on the duration of use

The specification of a defined duration of intake is not legally binding for vitamin A-containing dietary supplements, neither in Germany nor in the USA. In order to prevent an overdose with long-term intake, clear guidelines would be useful here depending on the dosage of the preparations.

### Differences between preparations from German and US online shops

Table 2 again compares the properties of the preparations from Germany and the USA. Despite largely similar legal requirements and recommendations in Germany and the USA, there are significant differences in the results between the various groups of preparations.

**Table 2:**
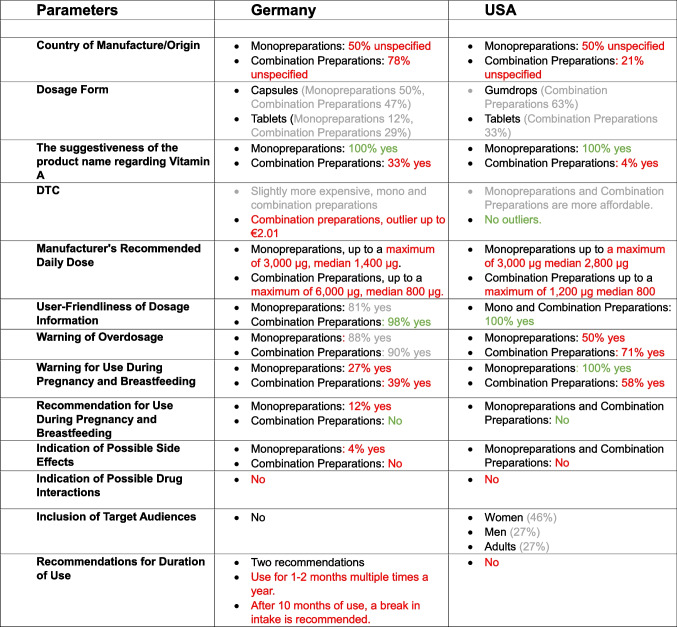
Comparison of the properties of German and US vitamin A preparations in tabular form. Green indicates positive evaluation by the authors; gray indicates ambivalent evaluation by the authors, and red indicates negative evaluation by the authors

The indication of the country of manufacture/country of origin is more reliable for the US preparations than for the German ones. The preference for gum drops in the US preparations is striking with regard to the dosage forms. With regard to the suggestiveness of the preparation names, the monopreparations show the same ratios, as they all have suggestive names. In comparison to the US-American combination preparations, German combination preparations are much easier to identify as containing vitamin A.

The daily therapy costs are somewhat higher for German combination preparations than for the US-American ones, and there are some outliers. The monopreparations are roughly on a par. The recommended daily dosages are significantly closer to the dosage recommendations for the US combination preparations than for the German ones, which is positive for consumer protection. The user-friendliness of the dosage information is complete for the US preparations and almost complete for the German preparations.

The warning against overdose is given in almost 90% of the German preparations, while only just under 70% of the US preparations give this warning. With regard to the warning against taking during pregnancy and breastfeeding, the US preparations (58%) are significantly better labeled than the German preparations (35%). In contrast to some German preparations (4%), the US-American preparations do not give a recommendation for use during pregnancy and breastfeeding. Only one of the German products provides information on possible side effects, while none of the US products do. Neither German nor US preparations contain any information on possible interactions.

## Limitations

The present results are based exclusively on the information and data obtained from the product descriptions and the outer packaging of the preparations. Due to the large number of vitamin A products available in online pharmacies as well as in pharmacies, health food stores, drugstores, and supermarkets in Germany and the USA, it was not possible to include all available products in this study. This limits the generalizability of the results. Larger studies, ideally including all products available in Germany and the USA, would therefore be desirable.

With regard to the adverse effects of vitamin A, although a recommended UL has been defined, the studies on teratogenicity, the negative influence on bone metabolism, and the possible negative influence on the risk of lung cancer in smokers are partly contradictory and do not yet allow a precise definition of an exact threshold dose.

## Conclusion

The preparations available in the USA and Germany differ significantly in some respects. Many of the vitamin A preparations and vitamin A-containing combination preparations do not fulfill the legal requirements for warning labels. In addition, manufacturers very often do not follow the recommendations of the BfR and the FDA in their dosage recommendations and for the most part offer preparations whose dosages significantly exceed the recommended amount for vitamin A in dietary supplements and the recommended daily intake, and in some cases even reach or exceed the UL. In addition, very high fluctuations in the ingredients of dietary supplements are tolerated, which can lead to unintentional under- or overdoses and limit consumer safety.

There is also a lack of adequate warnings for particularly vulnerable consumer groups such as pregnant women. Possible interactions between medicines and vitamin A are not specified but would also be very useful in view of demographic change and the frequent (poly)medication of many consumers. Recommendations on the duration of use only exist in exceptional cases. Only one preparation lists possible adverse effects/signs of an overdose although many preparations far exceed the recommended daily intake and the amount of vitamin A recommended for dietary supplements. This means that the dosage selected by the manufacturer already results in a vitamin A intake that exceeds the daily intake recommended by the BfR and the FDA.

Due to the enormous variety of preparations, it is easy for the consumer to lose track and possibly take several preparations containing vitamin A, as the names of the combination preparations are often not suggestive of vitamin A as an ingredient. The lack of information on the country of manufacture can also lead to uncertainty among consumers regarding the quality of the preparations. The wide range of DTCs also raises the question of whether a price regulation similar to the German Drug Price Regulation (AMPreisV 2023) should be considered for dietary supplements. In summary, it can be stated that there are clear deficits in compliance with the labeling requirements for the preparations and that the dosages are often questionable in view of the expert recommendations. Overall, these deficiencies can pose a risk for consumers that is difficult to assess in its entirety, especially for vulnerable consumer groups.

It is also clear that the problem of inadequate control and regulation of dietary supplements is not limited to specific products. In a recent research work (Trabert and Seifert 2024), it became clear that there are also considerable deficits in the area of ginkgo supplements. The study came to the conclusion that compliance with quality standards is also inadequate here. These findings suggest that the problems identified with vitamin A supplements are not isolated but rather point to a general lack of stringent regulation and monitoring in the dietary supplement sector.

If all preparations had to undergo an approval procedure before being launched on the market or alternatively had to comply with stricter requirements regarding labeling as containing vitamin A, the daily dosage, tolerance values of the concentration of the ingredients, naming of side effects and interactions and instructions for particularly vulnerable consumer groups, vitamin A preparations, and vitamin A-containing combination preparations could become safer for consumers.

Based on the available data and literature research, it is doubtful whether taking vitamin A supplements without a medically diagnosed vitamin A deficiency has a positive health benefit. Furthermore, it should be examined whether vitamin A should continue to be offered over-the-counter as a food supplement.

## Supplementary Information

Below is the link to the electronic supplementary material.

Supplementary file1 (DOCX 163 KB)

## Data Availability

All original data for this study are available from the authors upon reasonable request.

## Abbreviations

AMPreisV: Arzneimittelpreisverordnung (Pharmaceuticals Pricing Regulation)
BfR: Bundesinstitut für Risikobewertung (Federal Institute for Risk Assessment)
CFR: Code of Federal Regulations
DTC: Daily therapy costs
EAN: European Article Number
FDA: U.S. Food and Drug Administration
GTIN: Global Trade Item Number
NEM: Nahrungsergänzungsmittel (dietary supplements)
PZN: Pharmacy Central Number
SCCS: Scientific Committee on Consumer Safety
SCF: Scientific Committee on Food
TTK: Daily therapy costs (DTC)
UL: Tolerable upper intake level
UVP: Unverbindliche Preisempfehlung (suggested retail price (SRP))

## References

[CR1] Adolf T, W Heesche, Schneider R, Eberhardt W, Hartmann S, Herwig A, Heseker H, Hünchen K, Kübler W, Matiaske B, Moch KJ, Rosenbauer J, W Kübler, HJ Anders (1995) Ergebnisse der Nationalen Verzehrsstudie (1985-1988)¸ über die Lebensmittel- und Nährstoffaufnahme in der Bundesrepublik Deutschland. Wissenschaftlicher Fachverlag Dr. Fleck, Niederkleen

[CR2] Aktories K, Forth W (2013) Allgemeine und spezielle Pharmakologie und Toxikologie. Elsevier, München

[CR3] AMPreisV – Arzneimittelpreisverordnung (2023). https://www.gesetze-im-internet.de/ampreisv/BJNR021470980.html. Accessed 29 Nov 2023

[CR4] Arbeitskreis Nahrungsergänzungsmittel (AK NEM) im Lebensmittelverband Deutschland e. V. (2022) Pressemitteilung: Nahrungsergänzungsmittel – Absatz leicht rückläufig, Umsatz moderat gestiegen. https://www.lebensmittelverband.de/de/presse/pressemitteilungen/pm-20221018-nahrungsergaenzungsmittel-marktzahlen-2022. Accessed 7 Nov 2023

[CR5] Biesalski HK (1989) Comparative assessment of the toxicology of vitamin A and retinoids in man. Toxicology. 10.1016/0300-483x(89)90161-3 2665185 10.1016/0300-483x(89)90161-3

[CR6] Biesalski HK (2016) Vitamine und Minerale. Thieme Verlag, Stuttgart. 10.1055/b-0036-134841

[CR7] Brandt M (2022) Infografik: 3 von 4 Deutschen nehmen Nahrungsergänzungsmittel. Statista Daily Data. https://de.statista.com/infografik/24797/umfrage-zum-konsum-von-nahrungsergaenzungsmitteln-in-deutschland/. Accessed 7 Nov 2023

[CR8] Bundesinstitut für Risikobewertung (2021b) BfR-Verbrauchermonitor 2021 | Spezial Vitamine als Nahrungsergänzungsmittel. Bundesinstitut für Risikobewertung, Berlin

[CR9] Bundesinstitut für Risikobewertung (BfR) (2021a) Aktualisierte Höchstmengenvorschläge für Vitamine und Mineralstoffe in Nahrungsergänzungsmitteln und angereicherten Lebensmitteln (Stellungnahme Nr. 009/2021). 10.17590/20210315-143130. Accessed 8 Sep 2023

[CR10] Dawson IM (2000) The importance of vitamin A in nutrition. Curr Pharm Des 6(3). 10.2174/1381612003401190 10.2174/1381612003401190 10637381

[CR11] Domke A, Groflklaus R, Niemann B, Przyrembel H, Richter K, Schmidt E, Weiflenborn A, Wörner B, Ziegenhagen R (2008) Verwendung von Vitaminen in Lebensmitteln Toxikologische und ernährungsphysiologische Aspekte. BfR-Wissenschaft / Bundesinstitut für Risikobewertung, Berlin

[CR12] Europäische Kommission (2012). Verordnung (EU) 1169/2011. Dec 2012

[CR13] Europäische Kommission (2018). Durchführungsverordnung (EU) 2018/775. May 28, 2018

[CR14] Foot and Nutrition Board (FMB) Institute of Medicine (IOM) (2002) Dietary reference intakes for vitamin A, vitamin K, arsenic, boron, chromium, copper, iodine, iron, manganese, molybdenum, nickel, silicon, vanadium, and zinc. NAP.edu. 10.17226/10026 25057538

[CR15] Green AC, Kocovski P, Jovic T, Walia MK, RaS C, Martin TJ, Baker EK, Purton LE (2017) Retinoic acid receptor signalling directly regulates osteoblast and adipocyte differentiation from mesenchymal progenitor cells. Exp Cell Res. 10.1016/j.yexcr.2016.12.007 27964926 10.1016/j.yexcr.2016.12.007

[CR16] Hathcock JN, Hattan DG, Jenkins MY, McDonald JT, Sundaresan PR, Wilkening VL (1990) Evaluation of vitamin A toxicity. Am J Clin Nutr. 10.1093/ajcn/52.2.183 2197848 10.1093/ajcn/52.2.183

[CR17] Hayes WC, Cobel-Geard SR, Hanley TR, Murray JS, Freshour NL, Rao KS, John JA (1981) Teratogenic effects of vitamin A palmitate in Fischer 344 rats. Drug Chem Toxicol. 10.3109/01480548109018135 7338207 10.3109/01480548109018135

[CR18] Hendrickx AG, Peterson P, Hartmann D, Hummler H (2000) Vitamin A teratogenicity and risk assessment in the macaque retinoid model. Reprod Toxicol. 10.1016/s0890-6238(00)00091-5 10908834 10.1016/s0890-6238(00)00091-5

[CR19] Herbst EJ, Pavcek PL, Elvehjem CA (1944) Telang liver and vitamin A toxicity. Science. 10.1126/science.100.2598.338 17743365 10.1126/science.100.2598.338

[CR20] Kersting M, Alexy U, Kroke A, Lentze MJ (2004) Nutrition of children and adolescents. Results of the DONALD Study. Bundesgesundheitsblatt Gesundheitsforschung Gesundheitsschutz. 10.1007/s00103-003-0796-x 10.1007/s00103-003-0796-x 15205788

[CR21] Kowalski TE, Falestiny M, Furth E, Malet PF (1994) Vitamin A hepatotoxicity: a cautionary note regarding 25,000 IU supplements. Am J Med. 10.1016/0002-9343(94)90347-6 7985711 10.1016/0002-9343(94)90347-6

[CR22] Lind T, Öhman C, Calounova G, Rasmusson A, Andersson G, Pejler G, Melhus H (2017) Excessive dietary intake of vitamin A reduces skull bone thickness in mice. PLoS ONE. 10.1371/journal.pone.0176217 28426756 10.1371/journal.pone.0176217 PMC5398668

[CR23] NemV - Nahrungsergänzungsmittelverordnung (2004) https://www.gesetze-im-internet.de/nemv/NemV.pdf, Accessed 3 Nov 2023

[CR24] Nollevaux M, Guiot Y, Horsmans Y, Leclercq I, Rahier J, Geubel AP, Sempoux C (2006) Hypervitaminosis A-induced liver fibrosis: stellate cell activation and daily dose consumption. Liver Int. 10.1111/j.1478-3231.2005.01207.x 16448456 10.1111/j.1478-3231.2005.01207.x

[CR25] Omenn GS, Goodman GE, Thornquist MD, Balmes J, Cullen MR, Glass A, Keogh JP, Meyskens FL, Valanis B, Williams JH, Barnhart S, Cherniack MG, Brodkin CA, Hammar S (1996) Risk factors for lung cancer and for intervention effects in CARET, the Beta-Carotene and Retinol Efficacy Trial. J Natl Cancer Inst. 10.1093/jnci/88.21.1550 8901853 10.1093/jnci/88.21.1550

[CR26] Rothman KJ, Moore LL, Singer MR, Nguyen US, Mannino S, Milunsky A (1995) Teratogenicity of high vitamin A intake. N Engl J Med. 10.1056/NEJM199511233332101 7477116 10.1056/NEJM199511233332101

[CR27] Satia JA, Littman A, Slatore CG, Galanko JA, White E (2009) Long-term use of beta-carotene, retinol, lycopene, and lutein supplements and lung cancer risk: results from the VITamins and Lifestyle (VITAL) study. Am J Epidemiol. 10.1093/aje/kwn409 19208726 10.1093/aje/kwn409 PMC2842198

[CR28] Scientific Committee on Consumer Safety (SCCS) (2016) Opinion on vitamin A (retinol, retinyl acetate, retinyl palmitate) (SCCS/1576/16). https://ec.europa.eu/health/scientific_committees/consumer_safety/docs/sccso199.pdf. Accessed 29 Nov 2023

[CR29] Scientific Committee on Food (2002) Opinion on the tolerable upper intake level of preformed vitamin A (retinol and retinyl esters). SCF/CS/NUT/UPPLEV/24 Final, 7 October 2002. https://ec.europa.eu/food/fs/sc/scf/out145en.pdf. Accessed 29 Nov 2023

[CR30] Sporn MB, Roberts AB, Goodman DS (1971) The biochemistry of vitamin A. Academic Press, New York, NY

[CR31] Stickel F, Kessebohm K, Weimann R, Seitz HK (2011) Review of liver injury associated with dietary supplements. Liver Int. 10.1111/j.1478-3231.2010.02439.x 21457433 10.1111/j.1478-3231.2010.02439.x

[CR32] Trabert M, Seifert R (2024) Critical analysis of ginkgo preparations: comparison of approved drugs and dietary supplements marketed in Germany. Naunyn Schmiedebergs Arch Pharmacol. 10.1007/s00210-023-02602-6 37470803 10.1007/s00210-023-02602-6 PMC10771617

[CR33] U.S. Food and Drug Administration (2023a) CFR – Code of Federal Regulations Title 21 §101.5. https://www.accessdata.fda.gov/scripts/cdrh/cfdocs/cfcfr/cfrsearch.cfm?fr=101.5. Accessed 17 Oct 2023

[CR34] U.S. Food and Drug Administration (2023b) CFR – Code of Federal Regulations Title 21 §101.9. https://www.accessdata.fda.gov/scripts/cdrh/cfdocs/cfcfr/cfrsearch.cfm?fr=101.9. Accessed 17 Oct 2023

[CR35] U.S. Food and Drug Administration (2023c) CFR – Code of Federal Regulations Title 21 §101.9(g)(3) and (g)(4)). https://www.accessdata.fda.gov/scripts/cdrh/cfdocs/cfcfr/cfrsearch.cfm?fr=101.9. Accessed 17 Oct 2023

[CR36] U.S. Food and Drug Administration (2023d) CFR – Code of Federal Regulations Title 21 §101.9(g)(6). https://www.accessdata.fda.gov/scripts/cdrh/cfdocs/cfcfr/cfrsearch.cfm?fr=101.9. Accessed 17 Oct 2023

[CR37] U.S. Food and Drug Administration (2023e) CFR – Code of Federal Regulations Title 21 §101.17. https://www.accessdata.fda.gov/scripts/cdrh/cfdocs/cfcfr/CFRSearch.cfm?fr=101.17. Accessed 17 Oct 2023

[CR38] U.S. Food and Drug Administration (2023f) CFR – Code of Federal Regulations Title 21 §101.36(b)(2)(iii)). https://www.accessdata.fda.gov/scripts/cdrh/cfdocs/cfcfr/CFRSearch.cfm?fr=101.36. Accessed 17 Oct 2023

[CR39] U.S. Food and Drug Administration (2023g) CFR – Code of Federal Regulations Title 21 §201.63. https://www.accessdata.fda.gov/scripts/cdrh/cfdocs/cfcfr/CFRSearch.cfm?fr=201.63. Accessed 17 Oct 2023

[CR40] World Health Organization (2009) Global prevalence of vitamin A deficiency in populations at risk 1995–2005 World Health Organisation Global Database on Vitamin A Deficiency, Geneva http://whqlibdoc.who.int/publications/2009/9789241598019_eng.pdf. Accsessed 4 Dec 2023

[CR41] Yorgan TA, Heckt T, Rendenbach C, Helmis C, Seitz S, Streichert T, Amling M, Schinke T (2016) Immediate effects of retinoic acid on gene expression in primary murine osteoblasts. J Bone Miner Metab. 10.1007/s00774-015-0666-2 25956707 10.1007/s00774-015-0666-2

[CR42] Zhao T, Liu S, Zhang R, Zhao Z, Yu H, Pu L, Wang L, Han L (2022) Global burden of vitamin A deficiency in 204 countries and territories from 1990–2019. Nutrients. 10.3390/nu14050950 35267925 10.3390/nu14050950 PMC8912822

