# Supplementary material for: Vitamin A-containing dietary supplements from German and US online pharmacies: market and risk assessment
Source: Naunyn Schmiedebergs Arch Pharmacol. 2024 Mar 28;397(9):6803–20. doi: 10.1007/s00210-024-03050-6 (PMC11422271; doi:10.1007/s00210-024-03050-6)
Supplement: Supplementary file 1 — Supplementary file1 (DOCX 163 KB) [file 210_2024_3050_MOESM1_ESM.docx]

**Vitamin A-containing dietary supplements from German and US online pharmacies: Market and risk assessment**

**Anna-Miriam Rathmann and Roland Seifert**

**Supplemental Figures**

**
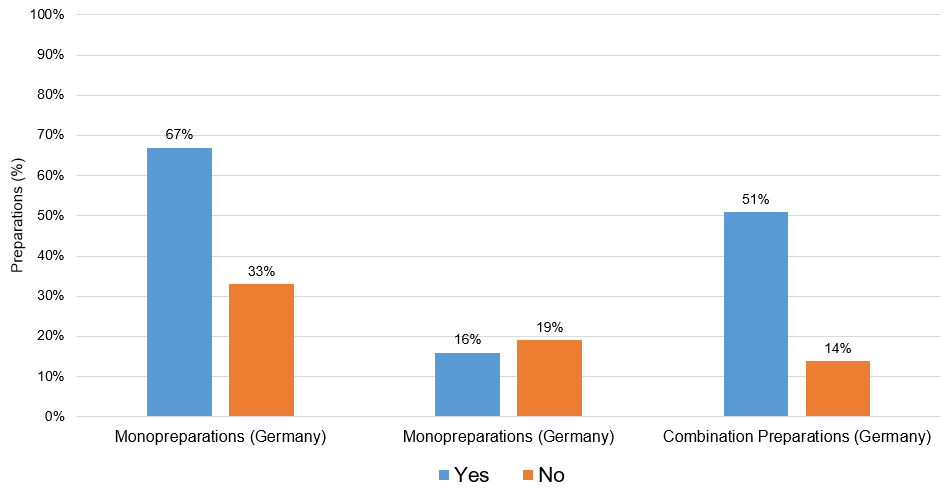
**

**Fig. S1:** Representation of the frequency of a daily dose equal to or less than the recommended intake of German preparations in the form of a bar chart, with the proportion of applicable preparations colored blue and the proportion of non-applicable preparations colored orange.


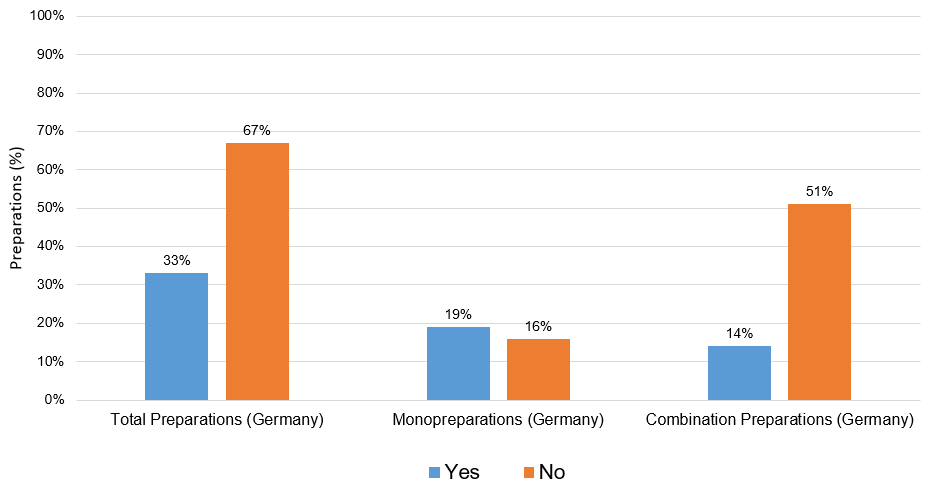


**Fig. S2:** Representation of the frequency of a daily dose above the recommended intake of the German preparations in the form of a bar chart, with the proportion of the respective applicable preparations colored in blue and the proportion of the non-applicable preparations colored in orange.


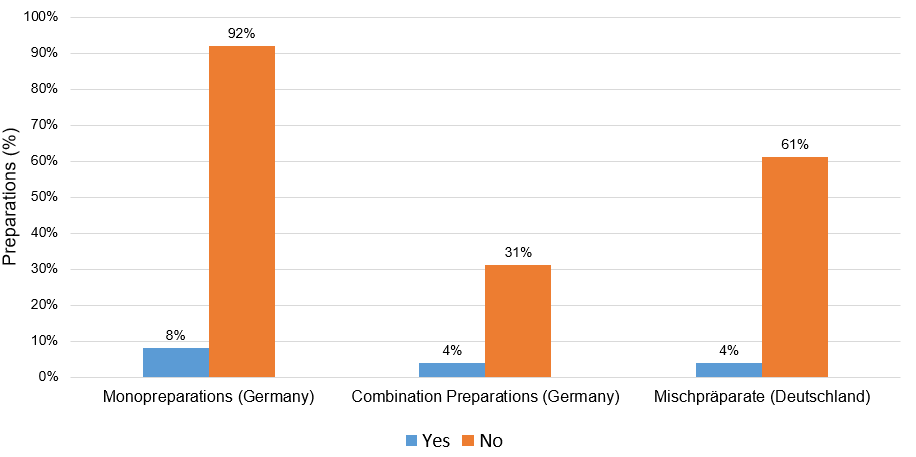
 **Fig. S3:** Representation of the frequency of daily doses equal to or above the UL of the German preparations in the form of a bar chart, with the proportion of applicable preparations colored in blue and the proportion of non-applicable preparations colored in orange.


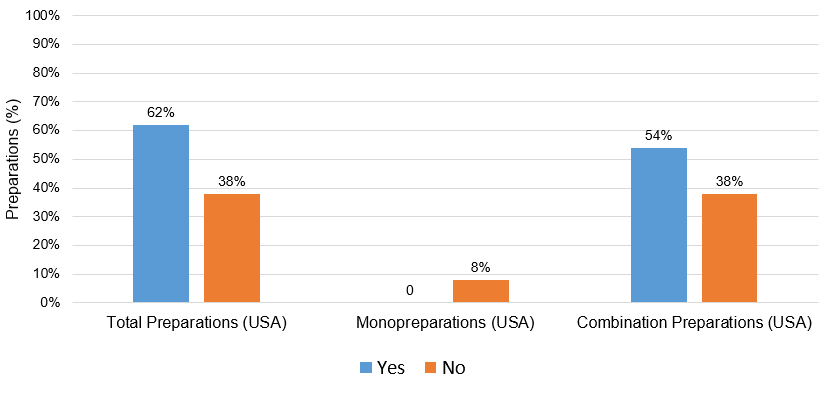
 **Fig. S4:** Representation of the frequency of a daily dose equal to or less than the recommended intake of the US preparations in the form of a bar chart, with the proportion of preparations that apply colored in blue and the proportion of preparations that do not apply colored in orange.


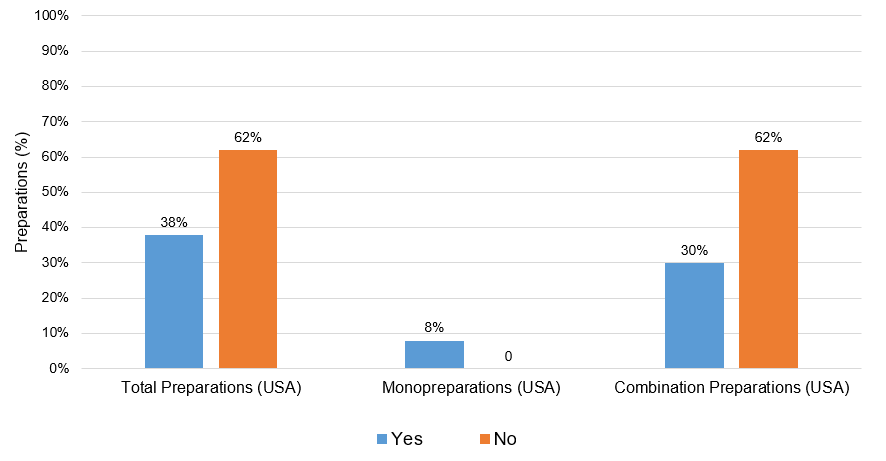

**Fig. S5:** Representation of the frequency of a daily dose above the recommended intake of U.S. drugs in the form of a bar chart, with the proportion of applicable drugs colored blue and the proportion of inapplicable drugs colored orange.


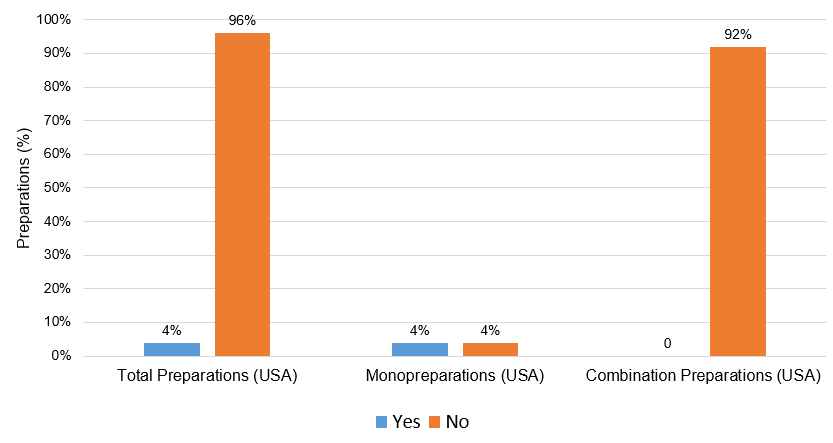

**Fig. S6:** The frequency of daily doses equal to or greater than the UL for U.S. products is presented as a bar chart, with the proportion of applicable products colored blue and the proportion of inapplicable products colored orange.


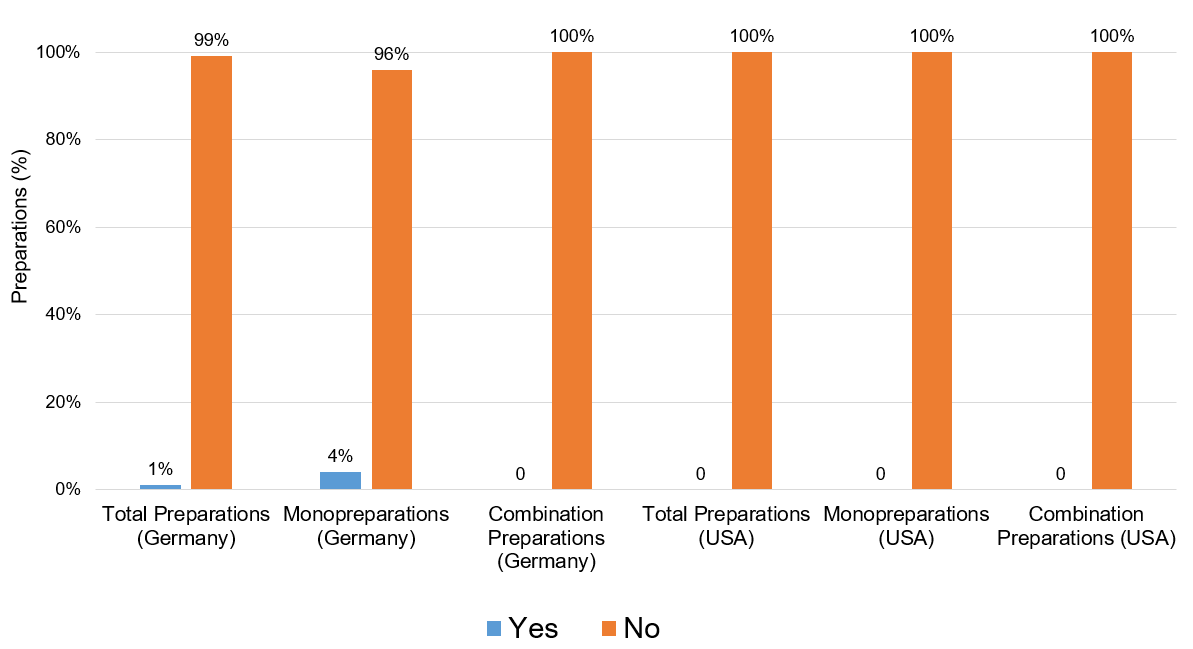


**Fig. S7:** Presentation of the frequency of a statement about possible adverse effects/side effects of the preparations in the form of a bar chart, where the proportion of preparations with a mention of possible adverse effects is colored blue and the proportion of preparations without a mention is colored orange.

**
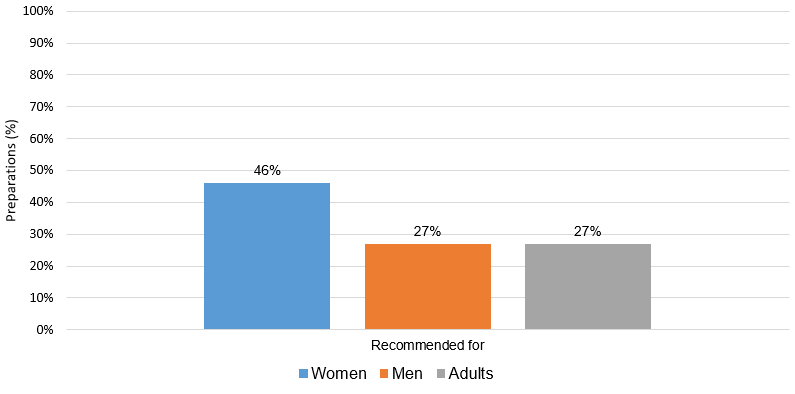
Fig. S8:** Frequency of target group mentions of U.S. products as a bar chart, with the proportion of products recommended for women in blue, the proportion of products recommended for men in orange, and the proportion of products advertised as neutral (for adults) in gray.
